# Comorbidities and Susceptibility to COVID-19: A Generalized Gene Set Data Mining Approach

**DOI:** 10.3390/jcm10081666

**Published:** 2021-04-13

**Authors:** Micaela F. Beckman, Farah Bahrani Mougeot, Jean-Luc C. Mougeot

**Affiliations:** Department of Oral Medicine, Carolinas Medical Center, Atrium Health, Charlotte, NC 28203, USA; Micaela.Beckman@atriumhealth.org

**Keywords:** SARS-CoV-2, COVID-19, comorbidity, SNP, susceptibility, severity

## Abstract

The COVID-19 pandemic has led to over 2.26 million deaths for almost 104 million confirmed cases worldwide, as of 4 February 2021 (WHO). Risk factors include pre-existing conditions such as cancer, cardiovascular disease, diabetes, and obesity. Although several vaccines have been deployed, there are few alternative anti-viral treatments available in the case of reduced or non-existent vaccine protection. Adopting a long-term holistic approach to cope with the COVID-19 pandemic appears critical with the emergence of novel and more infectious SARS-CoV-2 variants. Our objective was to identify comorbidity-associated single nucleotide polymorphisms (SNPs), potentially conferring increased susceptibility to SARS-CoV-2 infection using a computational meta-analysis approach. SNP datasets were downloaded from a publicly available genome-wide association studies (GWAS) catalog for 141 of 258 candidate COVID-19 comorbidities. Gene-level SNP analysis was performed to identify significant pathways by using the program MAGMA. An SNP annotation program was used to analyze MAGMA-identified genes. Differential gene expression was determined for significant genes across 30 general tissue types using the Functional and Annotation Mapping of GWAS online tool GENE2FUNC. COVID-19 comorbidities (*n* = 22) from six disease categories were found to have significant associated pathways, validated by Q–Q plots (*p* < 0.05). Protein–protein interactions of significant (*p* < 0.05) differentially expressed genes were visualized with the STRING program. Gene interaction networks were found to be relevant to SARS and influenza pathogenesis. In conclusion, we were able to identify the pathways potentially affected by or affecting SARS-CoV-2 infection in underlying medical conditions likely to confer susceptibility and/or the severity of COVID-19. Our findings have implications in future COVID-19 experimental research and treatment development.

## 1. Introduction

The COVID-19 pandemic’s first identified cases can be traced back to Wuhan, China, in December of 2019 [1]. As of 4 February, 2021 (WHO), there have been over 103.9 million confirmed COVID-19 cases affecting over 200 countries [2]. This staggering number of cases includes more than 2.26 million deaths, with the U.S. representing roughly one fourth of cases and deaths. A study at Stanford University estimated the infection fatality rate to be between 1.54 and 1.63%, which is significantly higher than the reported average mortality rate of 0.1% for influenza [3].

COVID-19 is caused by severe acute respiratory syndrome coronavirus-2, SARS-CoV-2. This highly pathogenic coronavirus can cause severe respiratory illness and is highly contagious. The incubation period for SARS-CoV-2 can last up to 14 days with a median range of 4 to 5 days from exposure to onset of symptoms [4]. Transmission of the infection is due to the inhalation of droplets or contact with contaminated surfaces. Symptoms include fever, cough, shortness of breath, fatigue, and body aches [5]. In addition to recently developed and deployed vaccines and monoclonal antibody therapies, treatment for severe SARS-CoV-2 illness includes the management of complications associated with the disease and supportive care [1,6]. There are currently few treatment options proven sufficiently effective for COVID-19 patients which complement vaccination strategies or ensure a milder course of the disease [7,8]. Thus, many drugs are being evaluated for effectiveness in reducing disease progression, severity, or mortality [7,8].

Multiple studies have demonstrated the influence of comorbidities on disease severity, quality of life, 1-year mortality, or altogether, in patients subject to viral infections [9,10]. Risk factors for the severity of SARS-CoV-2 infection include being aged 65 or above, and/or combined with a pre-existing condition [11,12]. Thus, patients with pre-existing cardiovascular disease, diabetes, kidney dysfunction, obesity, and pulmonary diseases may have worse clinical outcomes when infected with SARS-CoV-2 [13].

Understanding pathways which could determine the COVID-19 degree of susceptibility and severity are critical for drug development. Alternative drug combinations might act in synergy to complement vaccination strategies, because no vaccination provides absolute and indefinite protection [14]. Indeed, protection by vaccination against viral diseases may range from 9 to 90%, yet up to 60% of people vaccinated against influenza still fall ill due to the virus [15,16]. The current COVID-19 vaccines developed by Pfizer and Moderna have been reported to be nearly 95% effective at preventing severe disease and death [17,18]. However, vaccination may face some resistance among certain communities, is not systematically mandatory across countries, and presents a significant challenge for worldwide large-scale implementation. Moreover, novel emerging SARS-CoV-2 variants may evade vaccine protection over time [19].

Computational approaches may be utilized to identify candidate target molecules for the development of tailored drug treatments [8,20,21,22]. Drug targets can be predicted by gene expression or genetic polymorphism profile analysis using publicly available databases such as Gene Expression Omnibus (GEO) [23,24]. Computational tools may (i) help elucidate or compare drug mechanism(s) of action; (ii) assist in the identification and characterization of interactions between a drug and its target; and (iii) provide a better understanding of the mechanisms dictating cellular reactions to a drug response at the molecular level [25]. Furthermore, multiple overlapping genome-wide association studies (GWAS) have allowed researchers to determine the impact of single nucleotide polymorphisms (SNPs) on protein–protein interaction networks and the prediction of disease [26].

Computational approaches can serve in the investigation of viral susceptibility and contribute to the development of improved vaccines [27,28,29]. For instance, machine learning has been used in the development of viral vaccines (e.g., influenza) and in the investigation of genetic adaptation of the virus to the host [30]. Indeed, the genetic variability of SARS-CoV-2 will likely impact vaccine development in future outbreaks. Additionally, an appropriate computational model accounting for the complex network of molecular interactions between COVID-19 infection and various comorbidities would enhance the prediction and assessment of the mechanisms surrounding drug or vaccine treatment.

The aim of this study was to complete an SNP meta-analysis to identify genes associated with comorbidities/underlying medical conditions, potentially conferring increased susceptibility to SARS-CoV-2 infection or leading to the manifestation of severe viral symptoms. To this end, we conducted a generalized gene set analysis using single nucleotide polymorphisms (SNPs) data from genome-wide association studies (GWAS) of a comprehensive list of possible comorbidities using Multi-Marker Analysis of Genomic Annotation (MAGMA) [31]. This analysis was complemented with (i) the investigation of predicted effects from the significant SNPs identified by MAGMA and (ii) the determination of differential human gene expression, most likely relevant to the pathogenesis of the viral respiratory illnesses, severe acute respiratory syndrome (SARS) and influenza. A similar study has investigated host-interactome regulatory networks and pathway enrichment using publicly available data to identify the possible genes targeted by SARS-CoV-2 [32].

## 2. Methods

### 2.1. Multi-Marker Analysis of Genomic Annotation (MAGMA)

#### 2.1.1. GWAS Catalog and Gene Mapping

An initial list, representing 258 mostly chronic diseases, was determined using the Centers for Disease Control and Prevention (CDC) website information [33] and conventional literature searches grouped into 8 major disease categories (Data File S1). This list of 258 subcategories represented comorbidities/underlying medical conditions possibly associated with increased SARS-CoV-2 infectivity or disease severity. Due to the lack of published work for some subcategories, if the CDC stated that individuals with underlying heart conditions were at an increased risk for COVID-19, other cardiovascular conditions were included as subcategories. The intent of this list was not to represent all existing diseases, or all possible subcategories associated with the major categories, but to be representative of the available CDC information, overall, and to capture a broader repertoire of relevant GWAS data.

Using the initial list, SNP datasets from the online GWAS catalog database [34] were identified using the disease name. All associated SNPs were downloaded from the GWAS Catalog for each comorbidity and parsed to only include those with a *p*-value less than 0.05. SNPs from each comorbidity were mapped to genes separately using MAGMAv1.07b (CNCR, VU University, Amsterdam, The Netherlands) with the publicly available gene reference file NCBI37.3.gene.loc (https://ctg.cncr.nl/software/magma, accessed on 10 May 2020) containing 19,724 genes [31]. The locations of each SNP in each comorbidity file were mapped to a gene’s location from the gene reference file to produce an annotation file (https://ctg.cncr.nl/software/magma, accessed on 10 May 2020) [31].

#### 2.1.2. Determination of Multiple SNPs Significance

The significance of SNPs (*p*-values) and derived sample sizes pertaining to the genetic studies of comorbidities were extracted from the GWAS catalog datasets to compute correlations between neighboring genes and gene-level metrics via MAGMAv1.07b. [31]. To this end, the publicly available 1000 Genomes datasets (https://ctg.cncr.nl/software/magma, accessed on 10 May 2020) were used as reference files, considering the ethnicities associated with the possible comorbidity tested (European, East Asian, African, South American) [31]. MAGMAv1.07b was also used for gene-level analysis. To perform the gene-set SNP analysis, the ‘ncol’ flag was set to the to the sample size column in the SNP *p*-value file where each sample size corresponds to a *p*-value for the GWAS study completed. These values are used within MAGMAv1.07b to correct for the total number of samples tested in each comorbidity analysis. The flag for the “multi = all” model was used to perform the ‘linreg’, ‘mean’, and ‘top1′ model analysis. A Bonferroni-corrected significance threshold of *p* < 3.57 × 10^−4^ (0.05/140) was set for each comorbidity analyzed separately.

### 2.2. Pathway Analysis Using Enrichment Map and MAGMAv1.07b Programs

#### 2.2.1. Reactome Pathway Analysis

To conduct the pathway analysis, Reactome [35] human pathways were downloaded from Enrichment Map program [36] using Entrez gene IDs [37,38,39]. Computationally derived Gene Ontology (GO) biological terms and “No Data” were excluded. Based on the Bonferroni-corrected significant (*p* < 3.57 × 10^−4^) SNPs in comorbidity genes per MAGMAv1.07b analysis, the significant pathways also underwent a per-gene analysis with the MAGMAv1.07b model flag set to ‘alpha = 0.05’ where a gene-level matrix was computed with SNPs they contain as continuous variables. All analyses completed were one-sided, testing for positive associations. Associations of the genes with a phenotype is then calculated as a Z-score and transformed as a *p*-value to show whether genes in a gene-set are associated jointly with a phenotype [31]. The *p*-values of the genes used for downstream analysis were not corrected using a Bonferroni correction (threshold *p* < 2.53 × 10^−6^) but were corrected for multiple testing using the built-in automatic false discovery rate (FDR) cut-off function of MAGMAv1.07b (threshold *p* < 9.0 × 10^−6^). MAGMAv1.07b Intercept-only linear regression was then calculated for each gene set for further analysis.

#### 2.2.2. Interaction Networks

Visualization of protein–protein interaction networks was completed using STRINGv11.0 [40] program by testing different confidence levels to identify ontologies of biological significance for the significant pathways associated with comorbidities. A confidence level of 0.9 was used for all genes found significant by MAGMA to reduce the likelihood of false positives and to show the most probable interactions from the gene sets. A confidence level of 0.15 was also used to display the maximum number of protein–protein interactions possible for the VEP genes matched to MAGMA significant genes.

#### 2.2.3. Quality Control

Possible comorbidities significantly associated with gene sets/pathways were checked for quality control by generating quantile–quantile (Q–Q) plots using observed quantiles and residual Z-scores of genes within the gene set, based on the MAGMAv1.07b publicly available Rv3.6.2 script (posthoc_qc_107a.r, accessed on 20 July 2020) [41,42].

### 2.3. Prediction of SNP Effects

Ensembl’s Variant Effect Predictor program (VEP, European Bioinformatics Institute, Cambridge, UK) [43] was used to analyze MAGMAv1.07b annotation files for each gene set associated with comorbidities [44]. MAGMAv1.07b annotation files were converted into VEP format using a bash script. All converted annotation files were uploaded into VEP online tool separately. VEP summary statistics and analysis tables were downloaded for the 22 comorbidities’ associated genes and pathways found significant by MAGMAv1.07b. Corresponding tables were merged via Pythonv3.8.2 (Python Software Foundation, Fredericksburg, VA, USA) and SNPs containing a Sorting Intolerant from Tolerant (SIFT) score of 0 and a Polymorphism Phenotyping2 (PolyPhen2) score of 1, were removed (Data File S3). Human Genome Organisation (HUGO) gene symbols were extracted from the table with remaining SIFT and PolyPhen2 scores. Duplicate HUGO gene symbols were removed using Rv4.0.2 (R Foundation for Statistical Computing, Vienna, Austria). The most recently updated Affymetrix HG-U133A/B Human Genome Files [45,46] containing annotated gene symbols and Entrez gene identifiers for all human genes were used to retrieve missing gene identification [47]. These tabular (.csv) files were merged and loaded into Rv4.0.2. Entrez (National Center for Biotechnology Information (NCBI), Bethesda, MD, USA) gene IDs were matched to gene symbols from VEP analysis files to identify Affymetrix gene symbols. Genes and their corresponding Entrez ID’s were then matched to the significant genes’ Entrez IDs found through combined MAGMAv1.07b-STRING analysis (Institute of Molecular Life Sciences and Swiss Institute of Bioinformatics, Zurich, Switzerland).

### 2.4. Transcriptional Gene Expression Analysis

Functional Mapping and Annotation of Genome-Wide Association Studies (FUMA GWAS) GENE2FUNC online tool (https://fuma.ctglab.nl, accessed on 4 January 2021) was used to test the differential expression of genes based on MAGMAv1.07b (*n* = 119) and VEP (*n* = 50) significant genes [48]. All background genes were selected using “Ensemblv92” with “Genotype-Tissue Expression (GTEx)v8” representing “30 general tissues”. *p*-values were Benjamini–Hochberg corrected and set to a maximum of 0.05 with a minimum of two overlapping genes with gene sets [47]. Heatmaps were generated with GENE2FUNC online tool (VU University Amsterdam, Amsterdam, The Netherlands) using log2-transformed expression values clustering genes and tissue types.

### 2.5. Gene Involvement in Influenza and/or SARS

Significant genes of comorbidities’ associated pathways were compared to those found significant using GEO2R on a Gene Expression Omnibus (GEO) dataset for SARS (https://www.ncbi.nlm.nih.gov/geo/geo2r/?acc=GSE1739, accessed on 7 January 2021) [49]. Genes resulting from FUMA analysis that were highly upregulated in the lungs or blood, were used as input for STRINGv11.0 to confirm the possible comorbidities from significant Kyoto Encyclopedia of Genes and Genomes (KEGG) pathways. Furthermore, significant genes identified by MAGMAv1.07b (*n* = 119) were investigated to determine their roles in relation to influenza and/or SARS respiratory viral infections. Genes were cross-referenced using PubMed [50] literature searches, DisGeNETv6 (Integrative Biomedical Informatics Group, Barcelona, Spain) [51], Influenza Research Database [52] and GeneCodisv4.0 (Genyo Bioinformatics Unit, Granada, Spain) [53] including HUGO gene symbol and either “influenza” or “SARS” [54,55]. The risk of bias was assessed according to “Cochrane’s Handbook for Systematic Reviews of Interventions” [56]. Human tissue expression relevant to COVID-19 for genes with direct involvement was validated using Ensembl Expression Atlas [57,58]. Genes not generally expressed in the central nervous, cardiovascular, or pulmonary systems were removed from the dataset. Visualization of the protein–protein interaction network of genes directly involved with influenza and SARS (caused by SARS-CoV-1) was completed using STRINGv11.0 using an interaction score of 0.400 [40].

## 3. Results

**Figure 1:**
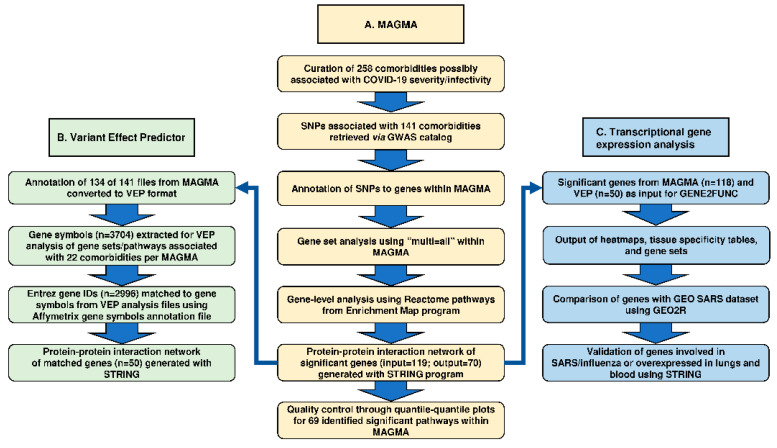
Computational analytical design for the determination of genes/pathways associated with comorbidities, possibly contributing to COVID-19 severity/infectivity. (**Section A**) A list of candidate comorbidities possibly associated with the increased severity/infectivity of COVID-19 were curated. Single nucleotide polymorphisms (SNPs) associated with curated comorbidities with available genome-wide association studies (GWAS) catalog data were analyzed separately. Multi-Marker Analysis of Genomic Annotation (MAGMA) was performed. SNPs were annotated to genes using NCBI gene reference file (NCBI37.3.gene.loc). In MAGMAv1.07b, gene set/pathway analysis was performed. Gene-level analysis was completed using Reactome pathways reference file retrieved from Enrichment Map program in MAGMAv1.07b. STRINGv11.0 protein–protein interaction program was used to visualize a network of 70 clustered genes. Quantile–quantile (Q–Q) plots in Rv3.4.2 were used for quality control. (**Section B**) MAGMAv1.07b annotation files were converted for Ensembl Variant Effect Predictor (VEP) format for 93% of datasets. Gene symbols were extracted for VEP analysis from the significant comorbidity-associated genes/pathways per MAGMAv1.07b analysis. Entrez gene IDs were matched to gene symbols using Affymetrix gene symbols annotation files (HG-U133A/B Human Genome Files). STRINGv1.0 protein–protein interaction program was used to visualize the network of significant genes. *(***Section C***)* Functional Mapping and Annotation of Genome-Wide Association Studies (FUMA) GENE2FUNC online tool was performed for 118 of 119 of MAGMAv1.07b identified genes. Differential gene expression (DEG) was visualized with a clustering of genes and tissue type. Significantly enriched DEG was determined and gene sets with at least two overlapping genes were identified. Gene Expression Omnibus (GEO) online tool, GEO2R (https://www.ncbi.nlm.nih.gov/geo/geo2r, accessed on 7 January 2021), was used to compare significant genes from a SARS dataset (GSE1739) with significant genes from MAGMAv1.07b. Genes overexpressed in tissue types “lung” and “blood” were entered into STRINGv11.0 to validate significant possible comorbidities from MAGMAv1.07b with significant Kyoto Encyclopedia of Genes and Genomes (KEGG) pathways. Tissue expression relevance to SARS and influenza was determined using DisGeNETv6 and Ensembl Expression Atlas databases.

The overall computational analytical design and associated primary results are presented in Figure 1.

### 3.1. MAGMA Analysis of Multiple SNPs Associated with Candidate COVID-19 Comorbidities

To conduct a generalized gene set analysis, we retrieved publicly available GWAS catalog datasets for 141 out of 258 COVID-19 possible comorbidities/underlying medical conditions (Data File S1). The 141 comorbidities were grouped into eight major categories by disease type, based on the organ most affected (Table S1). Following our MAGMA analysis (Figure 1A), of 141 comorbidities, MAGMAv1.07b was able to annotate SNPs to genes for each comorbidity input. Gene set analysis using MAGMAv1.07b then determined 5671 genes (3216 duplicates) to be significant (*p* < 3.57 × 10^−4^) from 140 comorbidities with chromosome 1 representing the highest number of unique genes and chromosome 6 representing the highest number of duplicates across all comorbidities (Table S2). Furthermore, roundabout guidance receptor 1 (ROBO1), BTB domain containing 9 (BTBD9), and teneurin transmembrane protein 3 (TENM3) all contained twenty or more SNPs used in analysis. The 140 merged output files with gene analysis results of the 140 comorbidities via MAGMAv1.07b are presented in Data File S2. Gene level analysis yielded 69 pathways which were derived from the 140 comorbidities analyzed separately. These pathways corresponded to 119 significant genes (*p* < 9.0 × 10^−6^) using MAGMAv1.07b automatic FDR correction, including 111 significant genes (*p* < 2.53 × 10^−6^) using Bonferroni correction. The pathways were significant for 22 COVID-19 comorbidities representing six disease categories, namely cancer (*n* = 9); cardiovascular (*n* = 4); neurologic/mental (*n* = 3); respiratory (*n* = 2); skin/musculoskeletal (*n* = 1); and autoimmune/endocrine/metabolic (*n* = 3).

**Figure 2:**
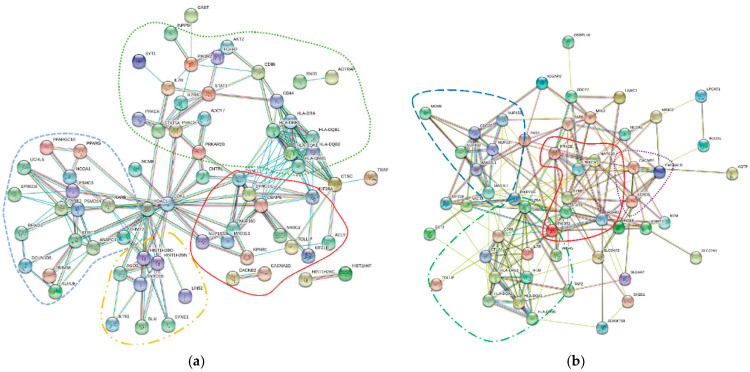
STRING protein–protein interaction network for significant COVID-19 comorbidity-associated genes. (**a**) MAGMA significant genes (CIS = 0.9). Significant genes were identified via MAGMAv1.07b gene-level analysis (https://ctg.cncr.nl/software/magma, accessed on 10 May 2020) using Reactome human pathways (https://reactome.org, accessed on 22 July 2020) obtained from Enrichment Map (http://baderlab.org/Software/EnrichmentMap, accessed on 22 July 2020) for 22 COVID-19 possible comorbidities with 119 significant genes. In STRINGv11.0 program, the confidence interaction score (CIS) was set to the maximum of 0.9, resulting in a network of 70 connected genes. Clustering by biological functions is represented by outlines. Green (…): cell regulation and immune response. Red (__): cell transport and nervous tissue function. Blue (--): protein homeostasis and gene expression. Orange (-..-): transcriptional regulation and RNA-mediated silencing. (**b**) Matched MAGMA and VEP significant genes (CIS = 0.15). Significant genes (*n* = 50) were identified for 22 COVID-19 possible comorbidities in both VEP SNP and MAGMAv1.07b gene-level analyses. CIS was set to low value of 0.150, which resulted in network integration of all 50 genes. Clustering by biological functions is outlined. Red (__): antigen-specific immune response. Blue (--): cell division and molecule formation/development. Green (--.--): cell growth, survival, proliferation, motility, and morphology. Purple (…): voltage gated ion channel transmembrane proteins. Note: Three gene symbols had other synonymous gene symbols (HLA-DRB1 and HLA-DRB5, HLA-DQA1 and HLA-DQA2, PIK3R2 and IFI30), which were also entered into STRINGv11.0 program for verification.

Reactome significant pathways and genes obtained through MAGMAv1.07b gene-level analysis from Enrichment Map are shown in Table 1 and Table 2. Using STRINGv11.0 program with the highest confidence interaction score (CIS) of 0.9, processing the 119 genes yielded a protein–protein interaction network of 70 genes, which was found to be highly significant based on hypergeometric test with Benjamini–Hochberg correction (*p* = 4.36 × 10^−11^) (Figure 2a). The top Kyoto Encyclopedia of Genes and Genomes (KEGG) pathway, identified by using STRINGv11.0, corresponded to the Epstein–Barr virus infection with FDR of 6.72 × 10^−9^.

Verification of the significant pathways using Q–Q plots showed a high association between genes and their relative gene ontology-defined pathways, since all plots show a distribution of residual *z*-scores deviating from the diagonal early on. There were no Q–Q plots with any ambiguous feature. Significant genes had high levels of association with each pathway. Q–Q plots of more than five genes, representing the pathway ontologies “post-translational protein modification”; “translocation of ZAP-70 to immunological synapse”; “metabolism”; and “cell cycle” and associated possible COVID-19 comorbidities (including asthma), are described in Figure S1.

### 3.2. VEP Analysis of MAGMA-Identified COVID-19 Comorbidity-Associated Genes

Annotation files were converted for 134 of the 141 comorbidities with GWAS catalog datasets available (Figure 1B). Of 3704 HUGO gene symbols extracted from VEP, 2996 corresponding Entrez gene IDs were identified using Affymetrix human genome annotation file. Of these gene IDs, 50 were matched with the 119 significant genes identified by MAGMAv1.07b for the 22 comorbidities with significant pathways (Table 3). Of the 50 genes, all were included in a protein–protein interaction network of 54 genes using a low CIS in STRINGv11.0 (Figure 2b). The top KEGG pathway identified using STRINGv11.0 was HTLV-1 infection with an FDR of 4.38 × 10^±7^ using a hypergeometric test with Benjamini–Hochberg correction.

### 3.3. Transcriptional Gene Expression Analysis of MAGMA- and VEP-Identified Genes

**Figure 3:**
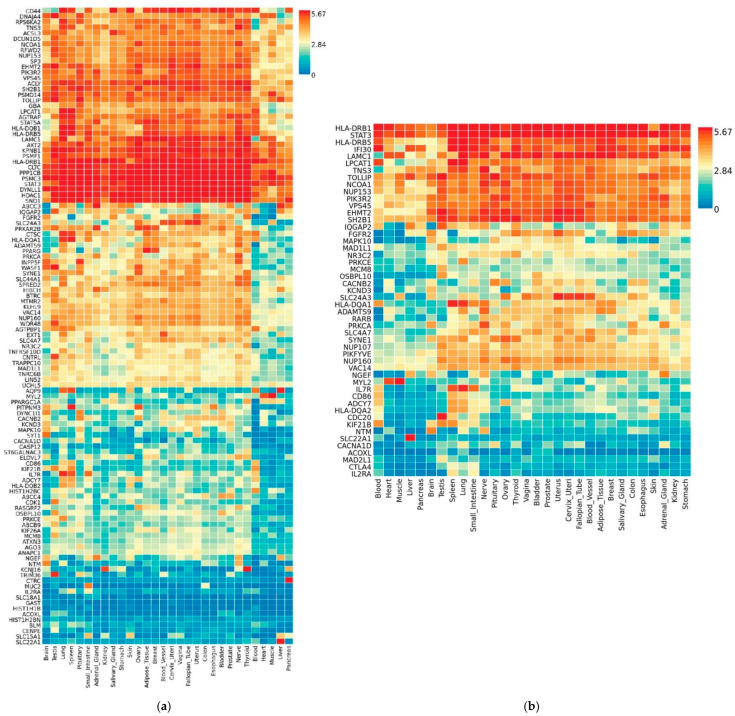
Human gene expression heatmap of MAGMA and VEP genes of COVID-19-associated comorbidities. Functional Mapping and Annotation of Genome-Wide Association Studies (FUMA GWAS) GENE2FUNC online tool was used to create a heatmap showing differential gene expression datasets using log2 transformed expression values from (**a**) Heatmap of MAGMAv1.07b significant genes. A total of 118 STRING significant genes were identified (*p* < 0.05) via MAGMAv1.07b and (**b**) the heatmap of 50 VEP matched genes from Ensembl Variant Effect Predictor (VEP) using (**a**,**b**) Genotype-Tissue Expression (GTEx) v8 representing 30 general tissue types. Red cells depict higher expression compared to cells filled in blue and yellow represents expression that is not significantly different from other genes.

FUMA GENE2FUNC was able to complete gene expression analysis for 118 of 119 and 50 genes found significant by MAGMAv1.07b with built-in FDR correction and VEP analysis, respectively. FUMA also identified 35,142 unique reference genes for the MAGMAv1.07b input. Heatmaps showing clustering of genes with 30 general tissue types are shown in Figure 3a,b. The tissue types, blood, heart, muscle, liver, and pancreas were among the 30 tightly clustered together. Results show the genes Lysophosphatidylcholine Acyltransferase 1 (LPCAT1), HLA Class II Histocompatibility Antigen, DR Beta 5 Chain (HLA-DRB5), Signal Transducer and Activator of Transcription 3 (STAT3), and HLA Class II Histocompatibility Antigen, DR1 Beta Chain (HLA-DRB1) were very upregulated in the significant tissue types, blood, and lungs. Results show Nucleoporin 160 (NUP160) had lower differential expression in the tissue types, blood, brain, liver, and heart for both MAGMAv1.07b input. Fibroblast Growth Factor Receptor 2 (FGFR2) was shown to have lower expression in tissue types: blood, heart, muscle, spleen, nerve, and adipose, from MAGMAv1.07b input (Figure 3a,b). Nucleoporin 153 (NUP153), Karyopherin Subunit Beta 1 (KPNB1), and Signal Transducer and Activator of Transcription 3 (STAT3) had higher expression compared to other genes in almost every tissue type (Figure 3a,b). Lower expression across nearly all tissue types included but was not limited to Interleukin 2 Receptor Subunit Alpha (IL2RA), Solute Carrier Family 18 Member A1 (SLC18A1), Gastrin (GAST), and Alpha-N-acetylgalactosaminide alpha-2,6-sialyltransferase 3 (ST6GALNAC3) from MAGMAv1.07b input (Figure 3a). GeneCodisv4.0 confirmed these genes, as well as others, to be involved in lung or viral biological processes. FUMA determined the significant upregulation of genes for the tissue types: blood and lung in MAGMAv1.07b input. Significant downregulation of genes for tissue types from MAGMAv1.07b input included liver, pancreas, kidney, breast, adrenal gland, testis, nerve, and muscle (Figure S2). The upregulation of genes expressed in the blood and lungs confirm asthma, and type I diabetes as possible comorbidities via significant STRINGv11.0 KEGG pathway results (data not shown). Gene sets with a minimum of two overlapping genes that match pathways identified using MAGMAv1.07b are shown in Table 2.

**Figure 4:**
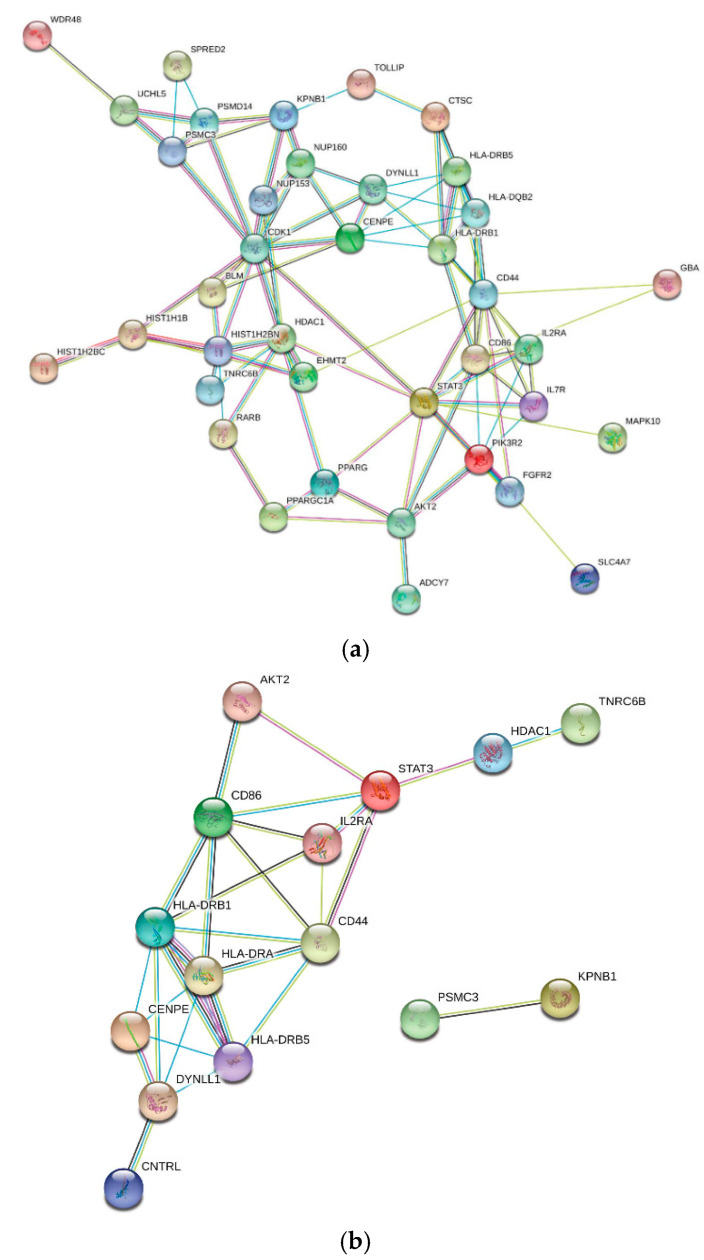
STRING protein–protein interaction of COVID-19 comorbidity-associated genes with involvement in influenza and/or SARS. STRING protein–protein interactions of MAGMAv1.07b identified genes with direct involvement with (**a**) influenza (CIS = 0.4). Influenza (*n* = 46) and/ or (**b**) SARS (CIS = 0.4). SARS (*n* = 17) are shown. Level of stringency in STRINGv11.0 program was set to a medium confidence interaction score (CIS) of 0.4 in both influenza and SARS-related molecular networks (**a**,**b**), resulting in a cluster of 38/46 (82.6%) and 15/17 (88.2%) genes, respectively.

The 119 genes analyzed for gene expression were also investigated for their possible role in influenza and SARS-CoV-1 infection, as these might be relevant to SARS-Cov-2 infection. Comparing significant genes from MAGMAv1.07b to a SARS GEO dataset analyzed by GEO2R, 92 of 119 genes were in common with 24 being a significant pre-Benjamini–Hochberg correction (data not shown). Of the 24, we identified 12 as being involved in SARS or influenza using conventional methods (Table S3). From MAGMAv1.07b, we identified three significant genes with a primary role in influenza infection: FGFR2, KPNB1 and NUP153 [59,60,61,62]. We also identified three genes KPNB1, Signal Transducer and Activator of Transcription 3 (STAT3), and Interleukin 2 Receptor Subunit Alpha (IL2RA) shown to play a significant role in SARS [63,64,65]. Genes identified as being possibly directly associated with influenza and/or SARS are shown in Table S3. STRING protein–protein interaction network yielded 38/46 (82.6%) genes involved in influenza and 15/17 (88.2%) genes involved in SARS, using an interaction score of 0.4 (Figure 4a,b). No GWAS study was found for SARS-CoV-1 infection to identify possible susceptibility genes within the 119 genes. However, genes overexpressed in blood and/or lungs for both MAGMAv1.07b and VEP input determined significant KEGG pathways for asthma and type I diabetes mellitus. Additionally, STRINGv11.0 concluded for both inputs that the genes upregulated in both tissue types belong to a local network cluster of Major Histocompatibility Complex (MHC) Class II protein complex (data not shown). No studies used for conventional searches were found to be at high risk for bias (Table S4).

## 4. Discussion

This is the first study conducting generalized gene set analysis on a broad spectrum of possible COVID-19 comorbidities, with the prospect of identifying comorbidity-specific genes that could impact infection by SARS-Cov-2. The assumption was made that, based on available CDC information, by including representative diseases/underlying conditions for eight major disease categories (Data File S1), we would analyze a broader and more informative set of GWAS data, and increase the likelihood to identify relevant gene expression signatures.

Thus, starting with a list of 258 diseases determined using CDC information, our MAGMA pipeline was able to identify 69 significant Reactome pathways with 119 significant genes using the automatic built-in FDR correction of MAGMAv1.07b, including 111 significant Bonferroni-corrected genes. The 119/111 genes represented 22 comorbidities from six disease categories that might have implications in predicting the severity of SARS-CoV-2 infection (Data File S1, Figure 1, Table 1, Table 2, Table S1) [33]. Of the 22 comorbidities, we were able to validate pathways associated with cardiovascular disease, diabetes, obesity, and pulmonary diseases. Cardiovascular diseases identified included heart failure, atherosclerosis, Kawasaki’s disease, and hypertension. Pulmonary diseases included asthma and interstitial lung disease. Cancer has been reported as a possible risk factor for COVID-19 [12]. We were able to identify nine cancers with GWAS data and significant associated pathways including acute myeloid leukemia, renal cell cancer, small cell lung cancer, and lung cancer. The known COVID-19 comorbidities—hypertension, obesity and diabetes—had significant pathways and genes.

While Q–Q plots indicated validity for 69 pathways (>5 genes) corresponding to six disease categories, caution for the interpretation of Q–Q plots must be used as these plots are normally used for pathways containing many genes. To a certain degree, these allow us to convey a certain level of confidence that there is a true association between the gene and pathway [42]. In our analysis, however, less genes identified allowed us to narrow possible gene targets and pathways. Indeed, certain genes identified in our study may have significant biological relevance to infection by SARS-COV-2. For instance, sialyl transferase ST6 N-acetylgalactosaminide alpha-2,6-sialyltransferase 3 (ST6GALNAC3) was found significant in the post-translational protein modification pathway (Figure S1). Another sialyl transferase, ST6GALNAC1, was previously investigated as a drug target against the infection of smooth airways epithelial cells by influenza virus [66]. It remains, however, to be determined whether ST6GALNAC3, generally expressed at high levels in renal cell cancer [67], plays a significant role in COVID-19 pathogenesis. STRINGv11.0 analysis produced significant enrichment for both MAGMAv1.07b genes and VEP matched genes containing SNPs that had characteristics of deleterious effects (Table 3). Therefore, we believe the interactions among the 119 genes from MAGMA and 50 matched VEP genes are likely not due to chance and that these genes are biologically connected. Furthermore, STRINGv11.0 analyses identified the top KEGG pathways including the Epstein–Barr virus pathway (MAGMA genes) and HTLV-1 pathway (VEP-matched genes). STRING was able to cluster 70 genes into four functional groups among the 119 MAGMA significant genes: cell regulation and immune response, cell transport and nervous tissue function, protein homeostasis and gene expression, and transcriptional regulation and RNA-mediated silencing (Figure 2a). Within the network of 70 genes, NUP160, NUP153, and KPNB1 clustered tightly together in the cell transport and nervous tissue function group.

STRINGv11.0 analysis of the 50 VEP matched genes with a lower confidence interval of 0.150 was required to obtain sufficient network connections for interpretation. Network analysis/interpretation may be subjective, dependent on pre-set confidence levels and established knowledge. It is, however, important to note that the enriched protein–protein interaction *p*-values were statistically significant. For the VEP matched gene STRINGv11.0 analysis, there were four distinct biological groupings recognized within the mapped network based on the closeness of protein interactions (Figure 2b). Those groupings were (i) antigen-specific immune response; (ii) cell division and molecule formation/development; (iii) cell growth, survival, proliferation, motility, and morphology, (iv) and voltage-gated ion channel transmembrane proteins. Notably, one of the comorbidities with significant associated pathways, breast cancer, contained SNPs affecting Solute Carrier Family 4 Member 7 (SLC4A7) and Solute Carrier Family 24 Member 3 (SLC24A3) genes. These genes are involved with sodium, calcium, and potassium ion transport and play a role in the malignant progression of breast cancer [68]. In addition, Euchromatic Histone Lysine Methyltransferase 2 (EHMT2) was mapped within close protein interactions. EHMT2 is involved with post-translational histone modification and epigenetic transcriptional repression. The orthologous gene (G9A) in drosophila is related to viral infection and susceptibility [69]. EHMT2 has been associated with the asthma comorbidity [70].

Heatmaps representing 118 MAGMA genes and 50 VEP genes across 30 general tissue types show the tissue types of blood, heart, muscle, and liver are tightly clustered (Figure 1C). Higher expression was seen across nearly all tissue types for the genes: HLA-DRB1, HLA-DQB1, KPNB1, NUP153, and STAT3 for both heatmaps (Figure 3a,b). FGFR2 was shown to have a very low expression in the tissue types of heart, muscle, liver, and spleen. Our analysis coincides with previous findings linking the induced inactivation of FGFR2 with increased mortality and influenza-induced lung injury [59]. Epithelial signaling by fibroblast growth factors is required for the effective recovery from lung injuries resulting from influenza infection [59]. EHMT2 was shown to have a high expression in nearly all tissue types except for blood, heart, muscle, liver, and pancreas. SLC24A3 shows a lower expression in the blood, heart, liver, and pancreas but there was no difference in expression compared to other genes in the tissue type of muscle. Furthermore, IL2RA had a low expression in nearly all tissue types (Figure 3a,b).

KPNB1 overexpression has been reported in several cancers including comorbidities we identified with significant genes and pathways (breast cancer, colorectal cancer, lung cancer, ovarian cancer, and prostate cancer) [71,72,73,74,75]. Overexpression in five of the nine tissue types observed in our heatmaps for KPNB1 and STAT3 may be due to having identified four additional cancer types as significant comorbidities (Figure 3a,b). Furthermore, KPNB1 is involved in the early stage of influenza virus replication via nuclear trafficking, by way of, nuclear import of viral cDNA or viral/host proteins into the host chromosome [60,61]. Both KPNB1 and NUP153 genes were found to be significantly upregulated in SARS with a logFC of 0.9, agreeing with the overexpression in our heatmap (Figure 3a) [62,63].

Based on previous studies, the interaction between NUP153 and KPNB1 has been investigated in relation to nuclear transport [76]. The degradation of NUP153 in influenza virus A-infected cells, such as Madin–Darby canine kidney II and human lung epithelial cells, results in an enlargement and widening of nuclear pores [62]. This disease process allows viral ribonucleoprotein complexes to be exported from the nucleus to the plasma membrane [62]. Additionally, NUP160 has been shown to work in conjunction with NUP153 to mediate nuclear import and export [77]. Therefore, the degradation of one or both can prevent the import of signal transducers and activators of transcription, reducing effectiveness of the anti-viral interferon response [78].

GeneCodis was able to identify FGFR2 as being involved in mesenchymal cell differentiation involved in lung development while NUP153 and NUP160 are involved in a viral replication process and intracellular transport of viruses. STAT3 was identified as being involved in primary miRNA binding and viral process and has been observed to be downregulated in SARS-CoV-1 infected Vero E6 kidney epithelial cells extracted from an African green monkey [64]. Additionally, IL2RA has been recently identified as significantly upregulated in the plasma of patients with severe COVID-19 [65] (Figure 2a,b). In our analysis, GeneCodis also identified Transporter 2, ATP binding Cassette Subfamily B Member (TAP2), Major Histocompatibility Complex, Class II, DR Beta 1 (HLA-DRB1), and Major Histocompatibility Complex, Class II, DQ Beta 1 (HLA-DQB1) as being involved in Epstein–Barr virus infection. Further in vitro and in vivo experimentation is needed to confirm these genes (or associated regulations) as possible drug targets for SARS-CoV-2 infection. Accordingly, this in silico analysis provides opportunities for researchers to explore new means to tackle the COVID-19 pandemic.

FUMA GENE2FUNC identified blood and lungs as having significantly upregulated differential gene expression (DEG) in both MAGMA and VEP inputs (Figure S2). Notably, lung and heart complications in those infected with SARS-CoV-2 are common [79]. STRING analysis identified genes upregulated in the blood and lungs for MAGMA and VEP inputs as having significant KEGG pathways for asthma and type I diabetes and a local network belonging to the MHC class II protein complex (data not shown). Asthma and type I diabetes were also identified by MAGMA as being significant comorbidities with significant pathways (Table 2). The four genes in common that were overexpressed in blood and lungs for both MAGMA and VEP inputs were Lysophosphatidylcholine Acyltransferase 1 (LPCAT1), Major Histocompatibility Complex, Class II DR Beta 5 (HLA-DRB5), Major Histocompatibility Complex, Class II DR Beta 1 (HLA-DRB1), and STAT3. LPCAT1 has been suggested as being essential for perinatal lung function and survival and surfactant homeostasis [80,81]. When LPCAT1 is overexpressed, the enzyme Carnitine palmitoyltransferase I (CPT1) is regulated in lung epithelial cells [81]. A knockdown of a subclass of this enzyme (CPT1-alpha) has been shown to inhibit Human Immunodeficiency Virus-1 replication [82].

Comparison of MAGMA significant genes with a SARS GEO dataset analyzed using GEO2R found 92 of 119 in common with 24 being significant prior to Benjamini–Hochberg correction (data not shown). Additionally, 71% of genes were in common with genes from the MAGMA string network. Interleukin Receptor 7 (IL7R) identified as significant in the multiple sclerosis possible comorbidity was shown to be significantly upregulated by 2.11 log-fold change (logFC) in SARS. IL7R can be found in B cells, T cells, and monocytes and is involved in the identity and defense of pathogens [83]. The heatmaps for MAGMA and VEP analysis show this gene to be overexpressed in the lungs and blood (Figure 3a,b). Other upregulated genes included SLC4A7, EHMT2, and Nuclear Receptor subfamily 3 group C member 2 (NR3C2) with logFCs of 2.33, 1.03, and 1.92, respectively. NR3C2 is a gene involved in the regulation of sodium levels and therefore blood pressure [83] which may confer susceptibility and severity of cardiovascular complications seen in patients with COVID-19. Significantly downregulated genes included Aquaporin 9 (AQP9) and Solute Carrier Family 22 Member 1 (SLC22A1) with logFCs of −2.06 and −1.95, respectively. Interestingly, Interleukin-7 (IL-7), encoded by IL7R, induces the expression of AQP9 [84]. Both IL7R and AQP9 were found to be overexpressed in the lungs by FUMA, AQP9 was under-expressed in the blood, and IL7R was overexpressed (Figure 3a,b). Thus, overall, we were able to identify many genes and pathways involving SNPs associated with comorbidities possibly altering gene expression and conferring increased susceptibility and/or severity of COVID-19. The findings of our study should be investigated further for their role of COVID-19.

## 5. Limitations

While there is no shortage of publicly available data, not all diseases have the same level of dedicated research. Therefore, not all possible comorbidities had publicly available SNP datasets from the GWAS catalog. This resulted in a large decrease from 258 possible comorbidities to 141. Furthermore, there is no standard to account for SNPs/genes with pleiotropic effects using MAGMAv1.07b. Another caveat is that FUMA GENE2FUNC uses mRNA expression datasets that have been generated through different independent studies using different analysis pipelines, so that the optimal normalization of raw data cannot be implemented.

The impact of many underlying conditions on COVID-19 infectivity, severity, or long-term consequences, is still unknown or the matter of current investigations. Additionally, there is unequal representation of GWAS data across diseases. Finally, there is limited knowledge about COVID-19 pathogenesis, although research on the matter has increased greatly since the beginning of the pandemic.

## 6. Conclusions

Significant pathways were identified associated with comorbidities/underlying medical conditions conferring susceptibility and/or severity to SARS-CoV-2 infection, which have been reported in conjunction with decreased clinical outcomes. Our findings may have implications in development of COVID-19 therapies.

## Figures and Tables

**Table 1:** Significant COVID-19 comorbidity-associated genes via MAGMAv1.07b. (a) SARS-CoV-2 infection-related possible comorbidity name having significant genes analyzed using MAGMAv1.07b (*n* = 22). (b) Entrez gene unique identifiers found to be significant (c) Gene symbol identified using DAVIDv6.8 (https://david.ncifcrf.gov/tools.jsp, accessed on 26 August 2020) from Entrez gene ID.* (d) p-value minimum and maximum; (e) p-value median; Comorbidities: (f) Cancer (non-head and neck cancer) group (*n* = 9), (g) Respiratory group (*n* = 2), (h) Cardiovascular/blood group (*n* = 4), (i) Neurologic/mental group (*n* = 3), (j) Autoimmune/metabolic/endocrine group (*n* = 3), and (k) Skin/musculoskeletal group (*n* = 1). Note: Bolded gene symbols (*n* = 50) represent matched genes from VEP analysis to significant genes found through MAGMAv1.07b analysis; Synonymous gene symbols include HLA-DRB1 with HLA-DRB5; HLA-DQA1 with HLA-DQA2; ADAMTS9 with PPARGC1A; IFI30 with PIK3R2.

Comorbidity ^a^	Entrez Gene ID/s ^b^	Gene Symbol ^c^	*p*-Value Min; Max ^d^	*p*-Value Median ^e^
Acute myeloid leukemia ^f^	3065; 256435; 51377; 6670; 5468; 57599; 56999; 10891; 4306; 9972; 1780; 55958; 23287; 1075; 84259; 50863; 4287; 123624; 641; 9491; 7109	HDAC1; ST6GALNAC3; UCHL5; SP3; PPARG; WDR48; PPARGC1A; **NR3C2**; **NUP153**; KLHL9; **AGTPBP1**; CTSC; DCUN1D5; **NTM**; ATXN3; AGBL1; BLM; PSMF1; TRAPPC10	1.53 × 10^−22^; 3 × 10^−6^	1.70 × 10^−10^
Asthma ^g^	55289; 2181; 79993; 47	**ACOXL**; ACSL3; ELOVL7; ACLY	6.26 × 10^−41^; 4 × 10^−7^	3.94 × 10^−24^
	2181; 79993; 47	ACSL3; ELOVL7; ACLY	1 × 10^−50^; 6.41 × 10^−9^	1.69 × 10^−10^
	2520; 5578; 6196	GAST; **PRKCA**; RPS6KA2	1 × 10^−50^; 6.41 × 10^−9^	3.21 × 10^−9^
	3122; 3127; 3117; 3119; 3120	HLA-DRA; **HLA-DRB5**; **HLA-DQA1**; HLA-DQB1; HLA-DQB2	1 × 10^−50^; 6.41 × 10^−9^	4.72 × 10^−24^
	2181; 79993	ACSL3; ELOVL7	1.72 × 10^−23^; 5.85 × 10^−8^	1 × 10^−8^
Atherosclerosis ^h^	6580; 6857	**SLC22A1**; SYT1	2 × 10^−43^; 1 × 10^−9^	5 × 10^−10^
	6580; 6564; 23446	**SLC22A1**; SLC15A1; SLC44A1	2 × 10^−43^; 2.67 × 10^−6^	7 × 10^−7^
	3773; 5577; 6580; 6857	KCNJ16; PRKAR2B; **SLC22A1**; SYT1	2 × 10^−43^; 1 × 10^−9^	1.2 × 10^−10^
	6580; 23446	SLC44A1; **SLC22A1**	2 × 10^−43^; 2.67 × 10^−6^	1.33 × 10^−6^
	6580; 23446; 23457; 5577; 6564	SLC44A1; **SLC22A1**; ABCB9; PRKAR2B; SLC15A1	2 × 10^−43^; 2.67 × 10^−6^	4 × 10^−7^
Bipolar disorder ^i^	11311; 23046; 26153; 25970	**VPS45**; **KIF21B**; KIF26A; **SH2B1**	1 × 10^−24^; 2 × 10^−6^	1 × 10^−6^
Breast cancer ^f^	64682; 983	ANAPC1; CDK1	1 × 10^−50^; 3 × 10^−8^	1.5 × 10^−8^
	983; 23112	CDK1; **TNRC6B**	1 × 10^−50^; 9.99 × 10^−35^	5 × 10^−35^
	64682; 983	ANAPC1; CDK1	1 × 10^−50^; 2 × 10^−9^	1 × 10^−9^
	23446; 57419; 9497	SLC44A1; **SLC24A3**; **SLC4A7**	9.58 × 10^−51^; 9 × 10^−6^	3 × 10^−45^
	57419; 9497	**SLC24A3**; **SLC4A7**	3 × 10^−45^; 9 × 10^−6^	4.5 × 10^−6^
Colorectal cancer ^f^	3915; 64759	**LAMC1**; **TNS3**	6.36 × 10^−14^; 2 × 10^−11^	1 × 10^−11^
	3915; 8936; 64759	**LAMC1**; WASF1; **TNS3**	6.36 × 10^−14^; 1 × 10^−6^	2 × 10^−11^
Heart failure ^h^	6570; 366	SLC18A1; AQP9	2 × 10^−44^; 2 × 10^−35^	1 × 10^−35^
Hypertension ^h^	57085; 27044	AGTRAP; SND1	4 × 10^−34^; 5 × 10^−7^	2.5 × 10^−7^
	3752; 776; 783; 4633	**KCND3**; **CACNA1D**; **CACNB2**; **MYL2**	1 × 10^−21^; 7 × 10^−12^	6.59 × 10^−16^
	3752; 776; 783	**KCND3**; **CACNA1D**; **CACNB2**	1 × 10^−21^; 1.31 × 10^−15^	1.08 × 10^−17^
	57085; 200734; 27044	AGTRAP; SPRED2; SND1	4 × 10^−34^; 5 × 10^−7^	2 × 10^−7^
Hypothyroidism ^j^	10213; 26275; 2131; 960; 8898; 113; 5296	PSMD14; HIBCH; **EXT1**; CD44; MTMR2; **ADCY7**; **PIK3R2**	3 × 10^−39^; 3 × 10^−10^	2 × 10^−17^
Interstitial lung disease ^g^	4583; 54472	**MUC2**; **TOLLIP**	7 × 10^−34^; 4.45 × 10^−13^	2.23 × 10^−13^
Kawasaki’s disease ^h^	55521; 6891; 208	TRIM36; **TAP2**; AKT2	5 × 10^−11^; 2 × 10^−8^	4 × 10^−10^
	55521; 6981	TRIM36; **TAP2**	5 × 10^−11^; 2 × 10^−8^	1 × 10^−8^
Lung cancer ^f^	374986; 79888; 22876	MIGA1; **LPCAT1**; INPP5F	8 × 10^−35^; 9 × 10^−6^	4 × 10^−7^
Multiple sclerosis ^k^	942; 5602; 3575; 3123; 3119	**CD86**; **MAPK10**; **IL7R**; **HLA-DRB1**; HLA-DQB1	6.08 × 10^−24^; 1 × 10^−11^	5 × 10^−20^
Obesity ^j^	25791; 5924	**NGEF**; RASGRF2	1 × 10^−50^; 5 × 10^−6^	2.5 × 10^−6^
	8648; 25791; 5915; 57698	**NCOA1**; **NGEF**; **RARB**; SHTN1	1 × 10^−50^; 8 × 10^−6^	2 × 10^−6^
	25791; 57698	**NGEF**; SHTN1	1 × 10^−50^; 8 × 10^−6^	4 × 10^−6^
	25791; 1062; 10788; 5924	NGET; CENPE; **IQGAP2**; RASGRF2	1 × 10^−50^; 8 × 10^−6^	2.5 × 10^−6^
Ovarian cancer ^f^	114884; 22876	**OSBPL10**; INPP5F	8 × 10^−35^; 2 × 10^−6^	1 × 10^−6^
Pancreatic cancer ^f^	2263; 6776; 6774	**FGFR2**; STAT5A; **STAT3**	1 × 10^−50^; 7 × 10^−6^	1 × 10^−6^
Prostate cancer ^f^	22876; 55697	INPP5F; **VAC14**	8 × 10^−35^; 2 × 10^−8^	1 × 10^−8^
	2629; 22876; 55697; 83394; 8714	GBA; INPP5F; **VAC14**; PITPNM3; ABCC3	1 × 10^−50^; 2 × 10^−6^	2 × 10^−8^
Renal cell cancer ^f^	5581; 8793	**PRKCE**; TNFRSF10D	1.5 × 10^−25^; 6 × 10^−9^	3 × 10^−9^
Schizophrenia ^i^	8294; 8341; 3009	HIST1H4I; HIST1H2BN; HIST1HIB	5 × 10^−27^; 2 × 10^−21^	9 × 10^−27^
	192669; 8294; 8341; 3009; 10919	AGO3; HIST1H4I; HIST1H2BN; HIST1H1B; **EHMT2**	5 × 10^−27^; 2 × 10^−6^	3.51 × 10^−19^
Small cell lung cancer ^f^	10919; 55466	**EHMT2**; DNAJA4	5 × 10^−21^; 5 × 10^−6^	2.5 × 10^−6^
Type 1 diabetes mellitus ^j^	10213; 3559	PSMD14; **IL2RA**	3.71 × 10^−31^; 4 × 10^−18^	2 × 10^−18^
Unipolar depression ^i^	64326; 5500; 8347; 8294; 8348; 23345; 8379; 11064; 8945; 5702; 23279; 8655; 91750; 3837	RFWD2; PPP1CB; HIST1H2BC; HIST1H4I; HIST1H2BO; **SYNE1**; **MAD1L1**; CNTRL; BTRC; PSMC3; **NUP160**; DYNLL1; LIN52; KPNB1	4 × 10^−25^; 7 × 10^−6^	6.75 × 10^−11^

**Table 2:** Reactome significant COVID-19 comorbidity-associated pathways from EnrichmentMap program via MAGMAv1.07b. (a) SARS-CoV-2 infection-related possible comorbidity name having significant pathways and genes analyzed using MAGMAv1.07b (*n* = 22). (b) Reactome pathway unique identifier; (c) Reactome pathway name, (d) p-value minimum and maximum; (e) p-value median; (f) Genes overlapping in Gene Sets via Functional Mapping and Annotation of Genome Wide Association Studies (FUMA GWAS) GENE2FUNC online tool (https://fuma.ctglab.nl/, accessed on 4 January 2021) results. Comorbidities: (g) Cancer (non-head and neck cancer) group (*n* = 9), (h) Respiratory group (*n* = 2), (i) Cardiovascular/blood group (*n* = 4), (j) Neurologic/mental group (*n* = 3), (k) Autoimmune/metabolic/endocrine group (*n* = 3), and (l) Skin/musculoskeletal group (*n* = 1).

Comorbidity ^a^	R-HSA Pathway ID ^b^	Reactome Pathways ^c^	*p*-Value Min; Max ^d^	*p*-Value Median ^e^	GENE2FUNC Overlapping Genes ^f^
Acute myeloid leukemia ^g^	597592	Post-translational protein modification	1.2 × 10^−4^	1.2 × 10^−4^	HDAC1, RFWD2, NCOA1, PSMD14, DYNC1I1
Asthma ^h^	1222499; 75105; 881907; 202433; 389948; 75876; 202430	Fatty acid metabolism; Fatty acyl-CoA biosynthesis and synthesis of very long-chain fatty acyl-CoAs; Gastrin-CREB signaling pathway via PKC and MAPK; Generation of second messenger molecules; PD-1 signaling; Translocation of ZAP-70 to immunological synapse	2.72 × 10^−10^; 6.93 × 10^−6^	2.27 × 10^−7^	HLA-DRA, HLA-DRB1;
Atherosclerosis ^i^	112310; 181430; 425407; 112315; 425366; 382551	Neurotransmitter release cycle & Norepinephrine neurotransmitter release cycle; SLC-mediated transmembrane transport; Transmission across chemical synapses; transport of bile salts and organic acids, metal ions and amine compounds, transport of small molecules	6.32 × 10^−9^; 4.18 × 10^−4^	4.36 × 10^−6^	ND
Bipolar disorder ^j^	983231	Factors involved in megakaryocyte development and platelet production	1.8 × 10^−6^	1.8 × 10^−6^	HDAC1, AGO3
Breast cancer ^g^	176814; 174048; 176409; 174143; 179419; 174048; 113507; 5687128; 176412; 176408; 453276; 425407; 425393	Activation of APC C and APC C: Cdc20 mediated degradation of mitotic proteins; Cyclin B; mitotic proteins; cell cycle proteins; cell cycle protein prior to satisfaction of cell cycle checkpoint; Phospho-APC C mediated degradation of Cyclin A; Phosphorylation and regulation of APC C between G1 S and early anaphase; E2F enabled inhibition of pre-replication complex formation; MAPK MAPK4 signaling; Regulation of mitotic cell cycle; SLC-mediated transmembrane transport; Transport of inorganic cations anions and amino acids oligopeptides	3.57 × 10^−11^; 3.32 × 10^−5^	1.34 × 10^−5^	ANAPC1, PSMD14, AGO3
Colorectal cancer ^g^	8875878; 6806834; 9006934	MET promotes cell motility; Signaling by MET and receptor tyrosine kinases	1.52 × 10^−4^; 5.67 × 10^−4^	3.6 × 10^−4^	ND
Heart failure ^i^	382551	Transport of small molecules	4.77 × 10^−5^	4.77 × 10^−5^	ND
Hypertension ^i^	5576891; 397014; 6802957; 6802952	Cardiac conduction; Muscle contraction; Oncogenic MAPK signaling	1.67 × 10^−6^; 3.03 × 10^−4^	3.70 × 10^−5^	ND
Hypothyroidism ^k^	1430728	Metabolism	3.08 × 10^−4^	3.08 × 10^−4^	HDAC1, NCOA1, PSMD14
Interstitial lung disease ^h^	168249	Innate immune system	6.06 × 10^−6^	6.06 × 10^−6^	CD44, PRKCE, PSMD14, HLA-DRA, HLA-DRB1
Kawasaki’s disease ^i^	1280218; 983169; 168256	Adaptive immune system & immune system; Class I MHC mediated antigen processing & presentation	8.02 × 10^−5^; 5.06 × 10^−4^	2.93 × 10^−4^	ANAPC1, PSMD14, CD86, TRIM36, HLA-DRA, HLA-DRB1, DYNC1I1
Lung cancer ^g^	1483257	Phospholipid metabolism	1.06 × 10^−4^	1.06 × 10^−4^	
Multiple sclerosis ^l^	1280215	Cytokine signaling in immune system	4.86 × 10^−5^	4.86 × 10^−5^	FGFR2, CD44, PSMD14, CD86, HLA-DRA, HLA-DRB1
Obesity ^k^	422475; 204998; 73887; 1266738; 416482; 9675108; 193648; 193704; 194840; 194315	Axon guidance; Cell death signaling via NRAGE, NRIF, and NADE; Death receptor signaling; Developmental biology; G alpha (12/13) signaling events; Nervous system development; NRAGE signals death through JNK; P75 NTR receptor-mediated signaling; Rho GTPase cycle; Signaling by Rho GTPases	5.78 × 10^−7^; 1.42 × 10^−4^	6.44 × 10^−7^	ND
Ovarian cancer ^g^	1483257	Phospholipid metabolism	9.76 × 10^−7^	9.76 × 10^−7^	ND
Pancreatic cancer ^g^	1226099	Signaling by FGFR in disease	2.4 × 10^−4^	2.4 × 10^−4^	ND
Prostate cancer ^g^	556833; 1483255; 1660516	Metabolism of lipids, PI; Synthesis of PIPs at the early endosome membrane	1.78 × 10^−5^; 8.64 × 10^−5^	5.21 × 10^−5^	ND
Renal cell cancer ^g^	109582	Hemostasis	1.71 × 10^−3^	1.71 × 10^−3^	ND
Schizophrenia ^j^	2559583; 2559586	Cellular senescence; DNA damage telomere stress induced senescence	1.16 × 10^−6^; 2.07 × 10^−6^	1.61 × 10^−6^	AGO3, ETS1, ANAPC1, EHMT2
Small cell lung cancer ^g^	8953897; 2262752	Cellular responses to external stimuli & stress	1.05 × 10^−3^	1.05 × 10^−3^	AGO3, ETS1, ANAPC1, PSMD14, EHMT2, DYNC1I1
Type 1 diabetes mellitus ^k^	4086398; 9607240; 5683057; 5673001; 8878171	ERK1 ERK2 pathway; FLT3 signaling, MAPK family signaling cascades; RAF MAP kinase cascade; Transcriptional regulation by RUNX1	2.77 × 10^−4^	2.77 × 10^−4^	HDAC1, AGO3, PSMD14
Unipolar depression ^j^	1640170	Cell cycle	9.12 × 10^−5^	9.12 × 10^−5^	HDAC1, RFWD2, ANAPC1, PSMD14, MCM8, DYNC1I1

**Table 3:** VEP genes and SNPs matched to significant MAGMAv1.07b COVID-19 comorbidity-associated genes. Total of 54 gene IDs from STRINGv11.0 analysis (50 genes after synonyms processed) were matched with significant genes from 22 comorbidities with significant pathways identified by MAGMAv1.07b. ^a^ Variant effect predictor (VEP) analysis of significant MAGMAv1.07b comorbidities; ^b^ Entrez gene unique identifiers found to be significant from STRINGv11.0 matched gene analysis; ^c^ Human Genome Organisation (HUGO) gene symbol identified using most recently updated Affymetrix HG-U133A/B Human Genome Files (http://www.affymetrix.com/Auth/analysis/downloads/na35/ivt/HG-U133A.na35.annot.csv.zip, http://www.affymetrix.com/Auth/analysis/downloads/na35/ivt/HG-U133B.na35.annot.csv.zip, accessed on 20 July 2020); ^d^ Single nucleotide polymorphism (SNP) identifiers of upload variant numbers from MAGMAv1.07b and VEP analysis (rs number); ^e^ Consequence of SNP variants on sequence; IV—intron variant; NMD—nonsense-mediated decay transcript variant; NC-T—noncoding transcript; NC-TV—noncoding transcript variant; 3prime—3 prime untranslated region variant; DGV—downstream gene variant; UGV—upstream gene variant; NC-EV—noncoding exon variant; IV-NC—intron variant, non-coding transcript; MS—missense variant; * Note: Comorbidities without significantly matched genes include mucocutaneous lymph node syndrome, renal cell cancer, and small cell lung cancer.

Comorbidity ^a^	Entrez Gene ID ^b^	Gene Symbol ^c^	Variant ID (rs#) ^d^	Consequence ^e^
Acute myeloid leukemia	2263; 5602; 55289; 56999; 9972; 50863	FGFR2; MAPK10; ACOXL; ADAMTS9; NUP153; NTM	7090018, 2912759; 6838659; 4640633; 17524344; 4849120; 4849121; 13395354; 9868005; 13095235; 4371513; 4605539; 11714364; 9851598; 4716165; 4716167; 10949435; 2274136; 9383307; 6906499; 9350055; 9396787; 10949436; 1006066; 11753865; 16879902; 12199222; 11222631; 11222631; 11222647; 12278021; 7107326; 11222652; 11222653; 992564; 12419920; 12575010; 4937627	IV; NMD; NC-TDGV; NC-TV; 3prime; MS
Asthma	5581; 3575; 3117; 3123; 6891; 3118; 10919; 56999	PRKCE; IL7R; HLA-DQA1; HLA-DRB1; TAP2; HLA-DQA2; EHMT2; ADAMTS9	12622534; 281508; 7717955; 6881270; 114798579; 146668528; 9272105; 3104369; 3104367; 9272346; 9270911; 2760995; 7760841; 4713555; 3997868; 151027268; 3104369; 3104367; 9272346; 41267086; 9866261	IV; IV, NC-TV; DGV; UGV; IV, NMD; 3prime; NC-EV
Atherosclerosis	114884; 50863	OSBPL10; NTM	1902341; 11827555	IV; IV, NC-TV
Bipolar disorder	25791; 783; 25970; 23345; 8379; 5578; 23046	NGEF; CACNB2; SH2B1; SYNE1; MAD1L1; PRKCA; KIF21B	778353; 2592118; 7071123; 3888190; 1203233; 17082664; 9371601; 7747960; 4523096; 4236274; 10275045; 4332037; 12668848; 3931398; 4721295; 1107592; 9895770; 2297909	IV; IV, NC-TV; IV, NMD; DGV; UGV
Breast cancer	9497; 57419; 23287	SLC4A7; SLC24A3; AGTPBP1	4973768; 7619833; 113118767; 77674461	3prime; DGV; IV, NC-TV; IV; IV, NMD
Colorectal cancer	4633; 3915; 64759; 57419; 2263	MYL2; LAMC1; TNS3; SLC24A3; FGFR2	17550549; 6678517; 4546885; 10911251; 3801081; 113118767; 11200014	IV; IV, NC-TV; DGV; IV, NMD
Heart failure	64759	TNS3	192154334	IV; DGV
Hypertension	776; 783; 84515	CACNA1D; CACNB2; MCM8	3774427; 12715461; 9814480; 12258967; 4815879	IV; IV, NC-TV; IV, NMD
Hypothyroidism	113	ADCY7	78534766	IV; DGV; MS, NMD; NC-EV; UGV
Interstitial lung disease	54472	TOLLIP	5743894; 5743890	IV; IV, NC-TV; UGV; IV, NMD
Lung cancer	79888; 8648	LPCAT1; NCOA1	4406174; 62140840; 11902506; 6710503	IV; IV, NMD; IV, NC-TV
Multiple sclerosis	5296; 6774; 3575; 942; 3117; 5602; 3118; 3559	PIK3R2; STAT3; IL7R; CD86; HLA-DQA1; MAPK10; HLA-DQA2; IL2RA	11554159; 2293152; 6897932; 10063294; 6881706; 2681424; 3104373; 2040406; 72665771; 3104373; 2040406; 2104286; 3118470; 12722489	DGV; IV; UGV; MS; NC-EV; IV, NMD; 3prime; IV, NC-TV
Obesity	5296; 10437; 25970; 5915	PIK3R2; IFI30; SH2B1; RARB	11554159; 7498665; 1435703	DGV; MS; NC-EV IV, NC-TV; UGV; IV, NC-TV; IV
Ovarian cancer	114884	OSBPL10	28568660	IV; IV, NC-TV; DGV
Pancreatic cancer	64759	TNS3	73328514	IV
Prostate cancer	55697; 64759; 3752; 6580; 8379; 23112; 2263	VAC14; TNS3; KCND3; SLC22A1; MAD1L1; TNRC6B; FGFR2	875858; 56232506; 2788612651164; 4646284; 527510716; 11704416; 9623117; 58133635; 12628051; 4821941; 11200014	IV; IV, NC-TV; IV, NMD; DGV; UGV
Schizophrenia	25791	NGEF	778371; 778353; 2944591	DGV; IV; UGV
Type 1 diabetes mellitus	3575; 3117; 3118; 3559	IL7R; HLA-DQA1; HLA-DQA2; IL2RA	6897932; 9272346; 927234661839660; 12722495; 706778; 10795791	MS; IV; UGV; NC-EV; NMD; NC-TV
Unipolar depression	25791; 783; 3123; 23345; 2131; 23279; 8379; 23046	NGEF; CACNB2; HLA-DRB1; SYNE1; EXT1; NUP160; MAD1L1; KIF21B	778353; 2799573; 7071123; 535777; 17082664; 9371601; 17506336; 11039409; 12668848; 1107592; 11514731; 2056477; 56072378; 3823624; 2297909	IV; NMD; NC-TV; UGV; DGV

## Data Availability

Publicly available data were used. Analyzed data are available upon request.

## Supplementary Materials

The following are available online at https://www.mdpi.com/article/10.3390/jcm10081666/s1, Figure S1. Q–Q Plots of possible COVID-19 comorbidity pathway-gene associations, Figure S2. Differential expression of genes by tissue specificity (a) regulation of DEG from MAGMAv1.07b significant genes by tissue type; and (b) regulation of DEG from VEP significant genes by tissue type, Table S1. Possible comorbidities associated with SARS-CoV-2 infectivity or disease severity, with available GWAS datasets, Table S2. Possible comorbidities associated with SARS-CoV-2 infectivity or disease severity, with available GWAS datasets, Table S3 Human COVID-19 comorbidity-associated genes involved in influenza and/or SARS pathogenesis, Table S4. Risk of bias assessment per PRISMA guideline, Data File S1. List of 258 mostly chronic diseases conferring possible susceptibility/severity of COVID-19 infection, Data File S2. Merged MAGMA significant gene results from 140 of 141 comorbidity output files, Data File S3. VEP results for significant comorbidities.
