# Supplementary material for: Comorbidities and Susceptibility to COVID-19: A Generalized Gene Set Data Mining Approach
Source: J Clin Med. 2021 Apr 13;10(8):1666. doi: 10.3390/jcm10081666 (PMC8070572; doi:10.3390/jcm10081666)
Supplement: Supplementary file 1 [file jcm-10-01666-s001.zip › Revised Suppl. Files/S1 Fig COVIDgenet QQ-plots 02-05-2021.pdf]

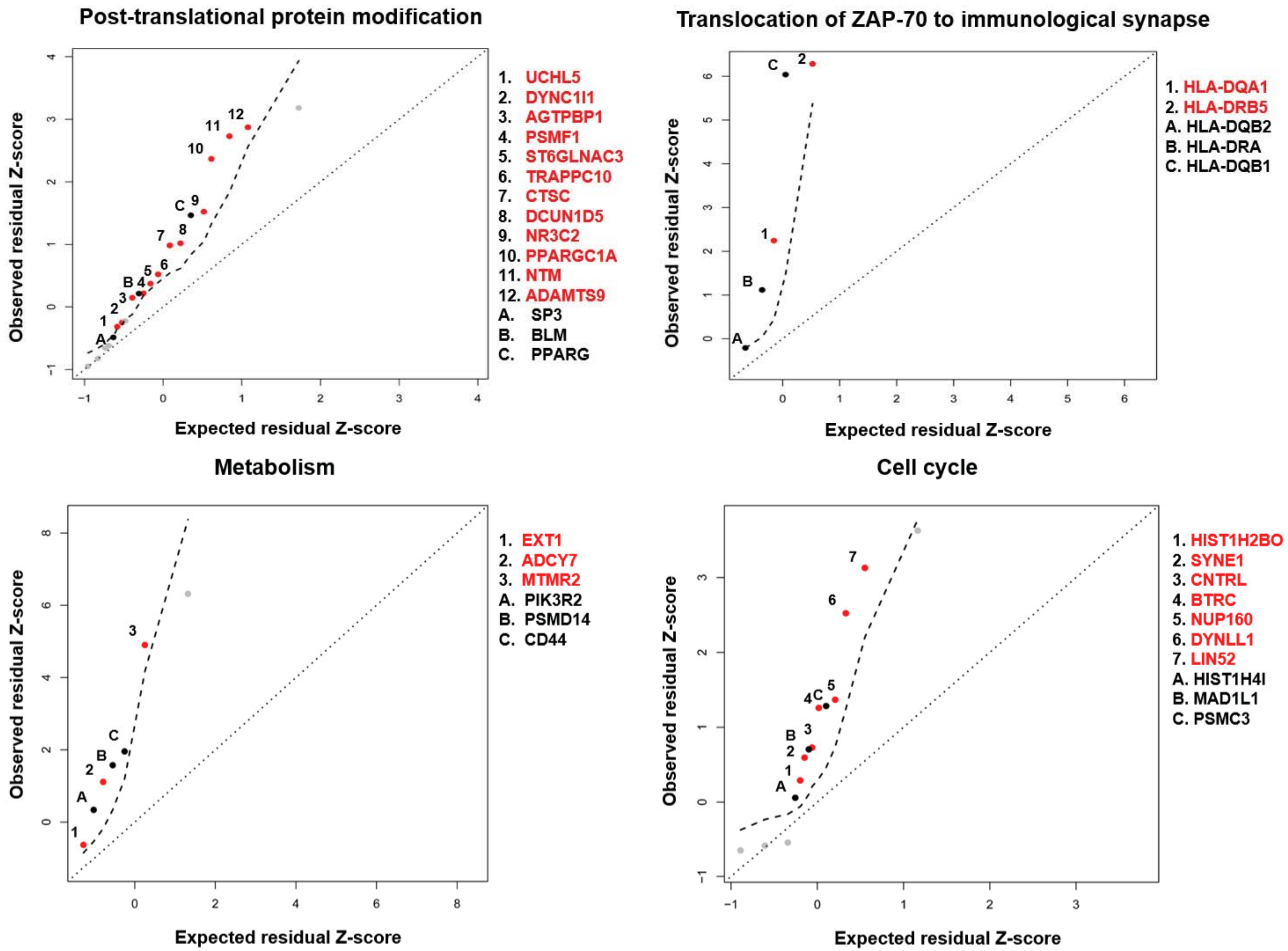

Post-translational protein modification
Observed residual Z-score
Expected residual Z-score
1. UCHL5
2. DYNC1I1
3. AGTPBP1
4. PSMF1
5. ST6GLNAC3
6. TRAPPC10
7. CTSC
8. DCUN1D5
9. NR3C2
10. PPARGC1A
11. NTM
12. ADAMTS9
A. SP3
B. BLM
C. PPARG
Translocation of ZAP-70 to immunological synapse
Observed residual Z-score
Expected residual Z-score
1. HLA-DQA1
2. HLA-DRB5
A. HLA-DQB2
B. HLA-DRA
C. HLA-DQB1
Metabolism
Observed residual Z-score
Expected residual Z-score
1. EXT1
2. ADCY7
3. MTMR2
A. PIK3R2
B. PSMD14
C. CD44
Cell cycle
Observed residual Z-score
Expected residual Z-score
1. HIST1H2BO
2. SYNE1
3. CNTRL
4. BTRC
5. NUP160
6. DYNLL1
7. LIN52
A. HIST1H4I
B. MAD1L1
C. PSMC3
