# Supplementary material for: Comorbidities and Susceptibility to COVID-19: A Generalized Gene Set Data Mining Approach
Source: J Clin Med. 2021 Apr 13;10(8):1666. doi: 10.3390/jcm10081666 (PMC8070572; doi:10.3390/jcm10081666)
Supplement: Supplementary file 1 [file jcm-10-01666-s001.zip › Revised Suppl. Files/S1 Fig COVIDgenet QQ-plots MB 04 08 21.docx]

**S1 Fig.** Q-Q plots of possible COVID-19 comorbidity pathway-gene associations

1.
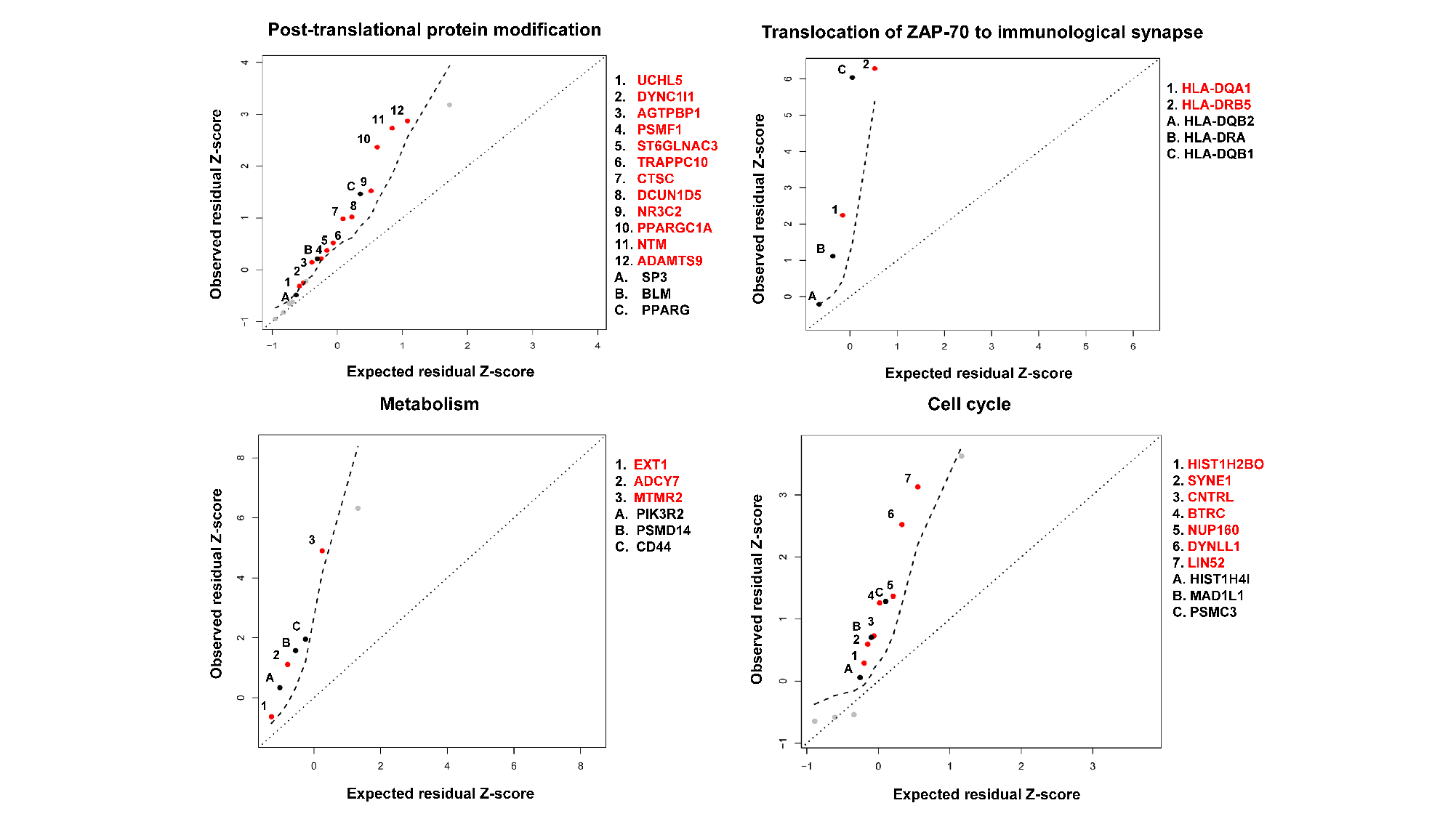
**Post-translational protein modification b. Translocation of Zap-70 to immunological synapse**

**
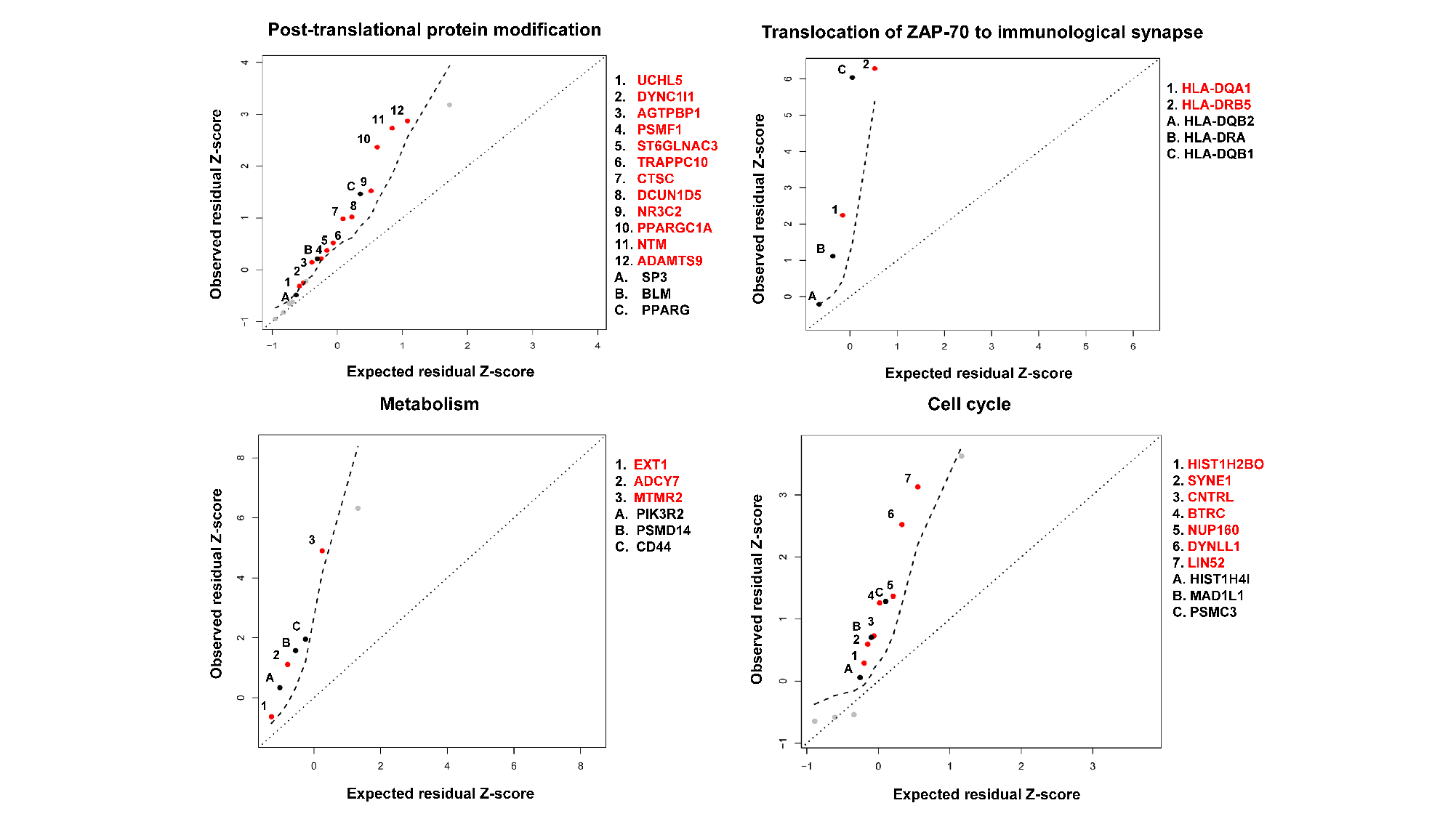
c. Metabolism d. cell cycle**

Quantile–quantile (Q–Q) plots of expected vs. observed residual z-scores of four possible comorbidities of coronavirus disease of 2019 (COVID-19) with significant pathways and genes using MAGMAv1.07b program and associated post hoc quality control R script are shown. The 95% upper confidence bands are based on all genes in the data using sample quantiles (**A.** 25%; **B.** 50%; **C.** 75%) of residual *z*-scores and depict the degree to which each gene’s z-score is likely to deviate from the expected value. Genes above the confidence band are shown in red while quantiles are shown in black. All genes below the confidence band are shown in gray. Gene symbols determined by DAVIDv6.8 (https://david.ncifcrf.gov/tools.jsp) from Entrez gene IDs correspond to labels shown for each plot. Gene symbols not shown are those below the confidence band. All Enrichment Map Reactome pathways (http://baderlab.org/Software/ EnrichmentMap) were identified using MAGMAv1.07b (https://ctg.cncr.nl/software/magma) from single nucleotide polymorphisms (SNPs) obtained at genome-wide association studies (GWAS) catalog online tool (https://www.ebi.ac.uk/gwas). For each possible comorbidity in each disease category, SNPs were annotated to genes and gene-level analysis was carried out where genes were identified as being associated with an Enrichment Map Reactome pathway. Out of 141 comorbidities, 22 comorbidities representing six disease categories had significantly associated gene-sets and genes within each set (*p* < 0.05). All gene-sets were found to have genes with a high level of association with each pathway. Q–Q plots pathway ontologies with 5 genes or more are shown. (**a**) Post-translational protein modification pathway associated with comorbidity “acute myeloid leukemia” from the “Cancer (non-Head and Neck Cancer)” disease category. (**b**) Translocation of zeta-chain-associated protein kinase 70 (*ZAP-70*) to immunological synapse pathway associated with the comorbidity “asthma” from the “Respiratory” disease category. (**c**) Metabolism pathway associated with COVID-19 comorbidity hypothyroidism from the “Autoimmune/Endocrine/Metabolic” disease category. (**d**) Cell cycle pathway associated with COVID-19 comorbidity unipolar depression from the “Neurologic/Mental” disease category. The number of genes per gene set was 21/1424 (**a**), 5/25 (**b**), 7/2098 (**c**), 14/682 (**d**), whereas the number of genes outside 95% confidence band was 14, 0, 6, and 10, respectively.
