# Supplementary material for: Comorbidities and Susceptibility to COVID-19: A Generalized Gene Set Data Mining Approach
Source: J Clin Med. 2021 Apr 13;10(8):1666. doi: 10.3390/jcm10081666 (PMC8070572; doi:10.3390/jcm10081666)
Supplement: Supplementary file 1 [file jcm-10-01666-s001.zip › Revised Suppl. Files/S2 Fig a&b COVIDgenet DEG tissues MB 04 08 21.docx]

**S2. Fig. Differential expression of genes by tissue specificity**

1. **Regulation of DEG from MAGMAv1.07b significant genes by tissue type**

**
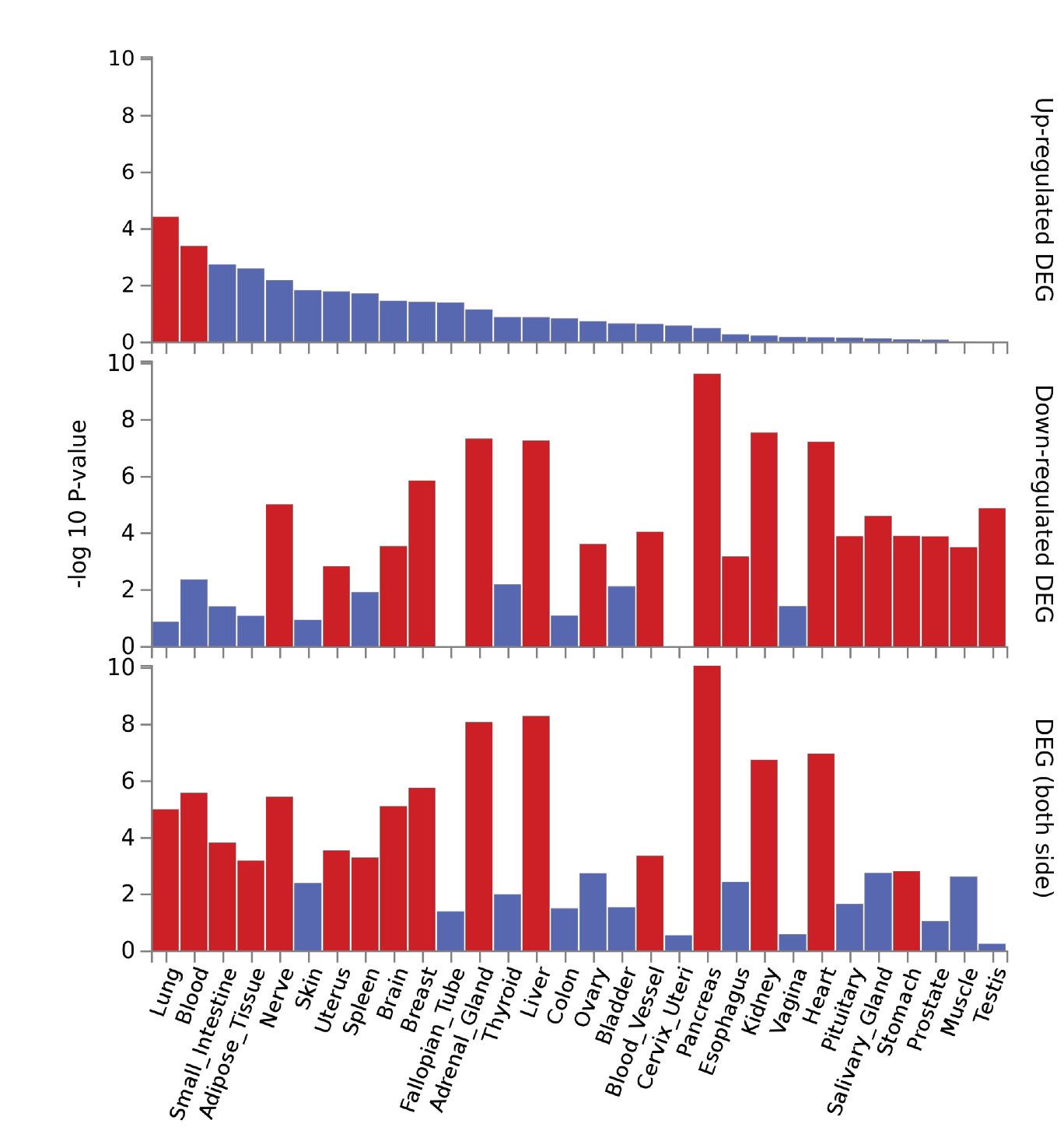
**

1. **Regulation DEG from VEP significant genes by tissue type**

**
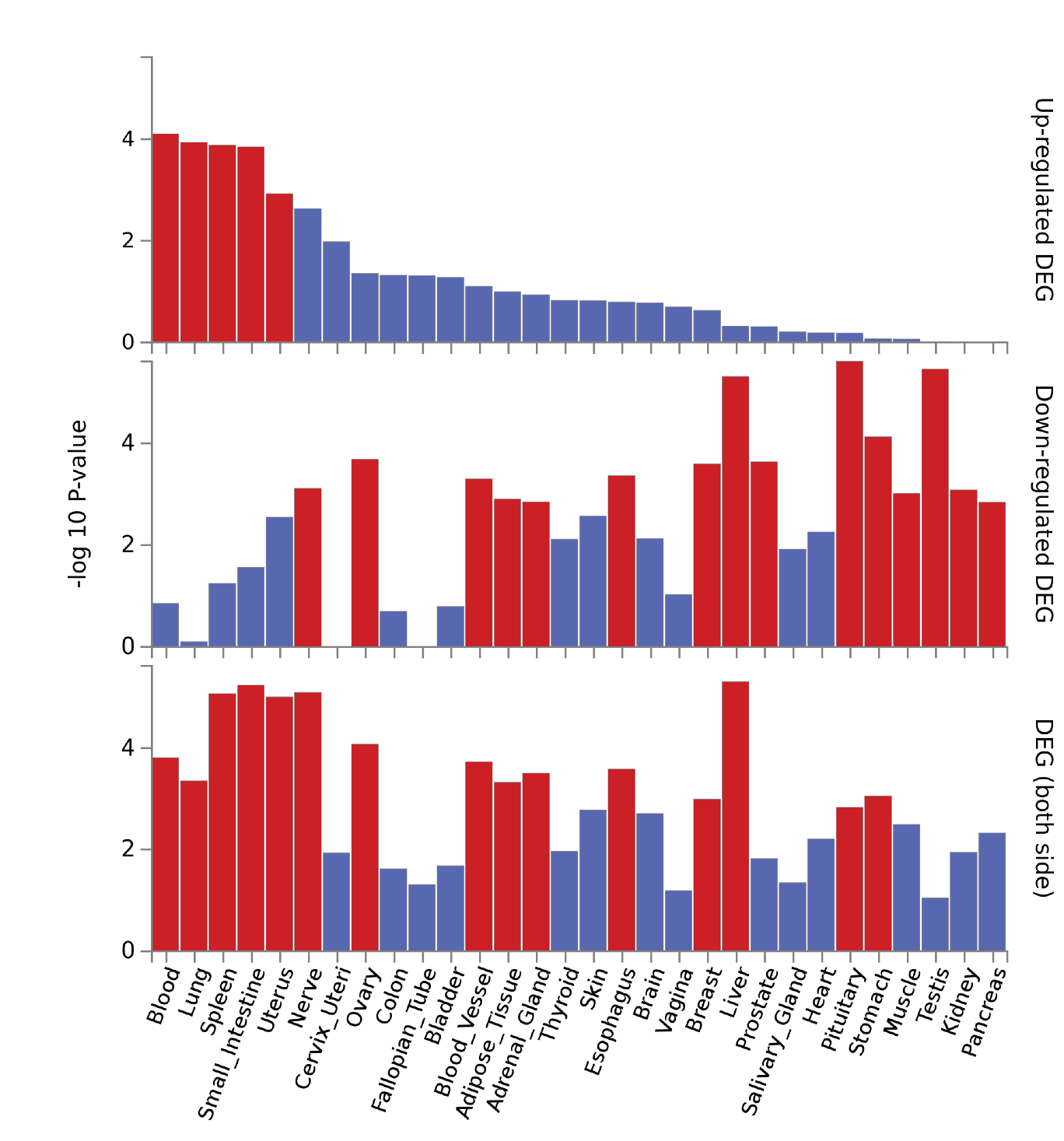
**

Differentially expressed genes by tissue type from GENE2FUNC Functional Mapping and Annotation of Genome-Wide Association Studies (FUMA GWAS) online program (https://fuma.ctglab.nl) in the order of upregulated DEG significance by performing a two-sided t-test for each label against all others for GTEx: v8 30 general tissue types. Input from (**a**) significant MAGMAv1.07b genes (*n* = 119) and (**b**) significant VEP genes (*n* = 50) were tested against each DEG set using a hypergeometric test. Significance is shown in red.
