# Supplementary material for: Comorbidities and Susceptibility to COVID-19: A Generalized Gene Set Data Mining Approach
Source: J Clin Med. 2021 Apr 13;10(8):1666. doi: 10.3390/jcm10081666 (PMC8070572; doi:10.3390/jcm10081666)
Supplement: Supplementary file 1 [file jcm-10-01666-s001.zip › Revised Suppl. Files/Table S1 COVIDgenet Comorbidities 04 08 21.docx]

| **Autoimmune/endocrine/ metabolic^a^** | **Cancer (non-HNC)^b^** | **Cardiovascular/blood^c^** | **Gastrointestinal/genitourinary^d^** | **Neurological/**  **mental^e^** | **Skin/**  **musculoskeletal^f^** | **Respiratory^g^** | **Viral/**  **bacterial^h^** |
| --- | --- | --- | --- | --- | --- | --- | --- |
| Autoimmune hepatitis | Acute lymphoblastic leukemia | Anemia | Alcoholic liver cirrhosis | Alcohol use disorder | Antiphospholipid antibody syndrome | **Asthma** | Hepatitis A |
| Diabetes mellitus | **Acute myeloid leukemia** | Aplastic anemia | Barrett’s esophagus | Anorexia nervosa | Atopic dermatitis | Atopic asthma | Hepatitis B |
| Hashitmoto’s thyroiditis | Bladder cancer | Aortic stenosis | Benign prostatic hyperplasia | Anxiety disorder | Dermatitis | Chronic bronchitis | Hepatitis C |
| Hyperthyroidism | **Breast cancer** | **Atherosclerosis** | Biliary liver cirrhosis | **Bipolar disorder** | Diffuse scleroderma | Cystic fibrosis | HIV/AIDS |
| **Hypothyroidism** | Cancer | Arrythmia | Celiac disease | Drug use disorder | Erythema multiforme | Emphysema | Shingles |
| **Obesity** | Cervical cancer | Cardiovascular disease | Chronic liver disease | Eating disorder | Gout | **Interstitial lung disease** | Vaginal yeast infection |
| Systemic scleroderma | Carcinoid tumor | Chagas cardiomyopathy | Chronic renal disease | Epilepsy | Limited scleroderma | Obstructive sleep apnea |  |
| Sjogren’s syndrome | Chronic lymphocytic leukemia | Aortic coarctation | Crohn’s disease | Generalized anxiety disorder | **Multiple sclerosis** | Pneumonia |  |
| **Type 1 diabetes mellitus** | Chronic myelogenous leukemia | Congenital heart malformation | Diverticular disease | Panic disorder | Osteoarthritis | Pulmonary embolism |  |
|  | CNS non-Hodgkin’s lymphoma | Congenital left-sided heart lesions | Gallstones | **Schizophrenia** | Osteoporosis | Pulmonary emphysema |  |
|  | **Colorectal cancer** | Dilated cardiomyopathy | Gastroesophageal reflux disease | **Unipolar depression** | Parkinson’s disease | Sarcoidosis |  |
|  | Cutaneous melanoma | **Heart failure** | IgA glomerulonephritis |  | Psoriasis | Sinusitis |  |
|  | Diffuse large-B-cell lymphoma | **Hypertension** | Kidney stones |  | Rheumatoid arthritis |  |  |
|  | Endometrial cancer | Hypertrophic cardiomyopathy | Membranous glomerulonephritis |  | Rosacea |  |  |
|  | Ewing sarcoma | Idiopathic dilated cardiomyopathy | Pancreatitis |  | Seborrheic dermatitis |  |  |
|  | Follicular lymphoma | Ischemic cardiomyopathy | Renal disease |  | Temporomandibular joint disorder |  |  |
|  | Gallbladder neoplasms | **Kawasaki’s disease** | Stomach/duodenal ulcers |  |  |  |  |
|  | Gastric cancer | Mitral valve prolapse | Stress urinary incontinence |  |  |  |  |
|  | Hodgkin’s lymphoma | Rheumatic heart disease | Ulcerative colitis |  |  |  |  |
|  | **Lung cancer** | Takotsubo cardiomyopathy | Urgency urinary incontinence |  |  |  |  |
|  | Lymphoma | Sickle cell anemia | Urinary incontinence |  |  |  |  |
|  | Male breast cancer | Stroke | Urinary tract infection |  |  |  |  |
|  | Malignant fibrosis histiocytoma | Tetralogy of Fallot |  |  |  |  |  |
|  | Melanoma |  |  |  |  |  |  |
|  | Metastatic colorectal cancer |  |  |  |  |  |  |
|  | Metastatic prostate cancer |  |  |  |  |  |  |
|  | Myeloproliferative neoplasms |  |  |  |  |  |  |
|  | Myelodysplastic syndrome |  |  |  |  |  |  |
|  | Non-small cell lung cancer |  |  |  |  |  |  |
|  | Osteosarcoma |  |  |  |  |  |  |
|  | **Ovarian cancer** |  |  |  |  |  |  |
|  | **Pancreatic cancer** |  |  |  |  |  |  |
|  | **Prostate cancer** |  |  |  |  |  |  |
|  | Rectum cancer |  |  |  |  |  |  |
|  | **Renal cell cancer** |  |  |  |  |  |  |
|  | **Small cell lung cancer** |  |  |  |  |  |  |

**S1 Table.** Possible comorbidities associated with SARS-CoV-2 infectivity or disease severity, with available GWAS datasets

Comorbidities possibly associated with increased infectivity or severity of coronavirus disease of 2019 (COVID-19) consisting of 8 categories and representing 141 of initially screened 258 diseases were analyzed using MAGMAv1.07b and Ensembl Variant Effect Predictor. Comorbidities shown in **bold** (*n* = 22) correspond to those with significant associated genes and pathways following Multi-Marker Analysis of Genomic Annotation (MAGMA). Disease, disorder, and infection types include: **^a^** Autoimmune/endocrine/metabolic (*n* = 9), **^b^** Non-head and neck cancers (non-HNC) (*n* = 37), **^c^** Cardiovascular/blood (*n* = 23), **^d^** Gastrointestinal/genitourinary (*n* = 22), **^e^** Neurologic/mental (*n* = 11), **^f^** Skin/musculoskeletal (*n* = 18), **^h^** Respiratory (*n* = 14), **^I^** Viral/bacterial (*n* = 8). Note: Diseases with multiple levels, progressions, or sites (e.g., chronic, acute, child onset, adult onset, knee, hand, etc.) are not shown for simplification.
