# Supplementary material for: Comorbidities and Susceptibility to COVID-19: A Generalized Gene Set Data Mining Approach
Source: J Clin Med. 2021 Apr 13;10(8):1666. doi: 10.3390/jcm10081666 (PMC8070572; doi:10.3390/jcm10081666)
Supplement: Supplementary file 1 [file jcm-10-01666-s001.zip › Revised Suppl. Files/Table S2. Total significant genes by chromosome MB JLM MB 04 08 21.docx]

| **Chromosome^a^** | **Total # of genes^b^** | **# unique genes^c^** | **% duplicates^d^** |
| --- | --- | --- | --- |
| 1 | 578 | 345 | 40.31 |
| 2 | 494 | 250 | 49.39 |
| 3 | 400 | 223 | 44.25 |
| 4 | 222 | 138 | 37.84 |
| 5 | 295 | 162 | 45.08 |
| 6 | 564 | 270 | 52.13 |
| 7 | 328 | 172 | 47.56 |
| 8 | 209 | 116 | 44.50 |
| 9 | 227 | 127 | 44.05 |
| 10 | 322 | 175 | 45.65 |
| 11 | 349 | 204 | 41.55 |
| 12 | 245 | 153 | 37.55 |
| 13 | 96 | 56 | 41.67 |
| 14 | 136 | 88 | 35.29 |
| 15 | 161 | 104 | 35.40 |
| 16 | 230 | 124 | 46.09 |
| 17 | 274 | 155 | 43.43 |
| 18 | 88 | 55 | 37.50 |
| 19 | 165 | 114 | 30.91 |
| 20 | 97 | 58 | 40.21 |
| 21 | 46 | 32 | 30.43 |
| 22 | 113 | 66 | 41.59 |
| X | 32 | 29 | 9.38 |
| **Total** | **5671** | **3216** | **43.29%** |

**S2 Table.** MAGMAv1.07b significant genes by chromosome from 140 of 141 possible comorbidities

Counts of significant (*p* < 2 × 10 − 4) genes determined from 140 of 141 possible comorbidities using MAGMAv1.07b. ^a^ Human chromosome gene location; ^b^ total number of significant genes; ^c^ number of significant genes with duplicates removed; ^d^ percentage of duplicate significant genes to the total (duplicate divided by total number of genes); # is number; % is percent. Note: the comorbidity “aortic coarctation” had no significant genes.
