# Supplementary material for: Comorbidities and Susceptibility to COVID-19: A Generalized Gene Set Data Mining Approach
Source: J Clin Med. 2021 Apr 13;10(8):1666. doi: 10.3390/jcm10081666 (PMC8070572; doi:10.3390/jcm10081666)
Supplement: Supplementary file 1 [file jcm-10-01666-s001.zip › Revised Suppl. Files/Table S3 COVIDgenet Influenza SARS 04 08 21.docx]

| **Gene^a^** | **Influenza^b^** | **SARS^c^** | **Cell type^d^** | **PMID/reference^e^** |
| --- | --- | --- | --- | --- |
| ACSL3 | Downregulation | Protein interaction | Cancer; Lung cancer | 32444466; IRD* |
| ADCY7 | Pro-viral | ND | Primary HBE | 20064372 |
| AGTRAP | Replication | ND | MDCK | 20027183 |
| AKT2 | Protein synthesis | Downregulation | MDCK, A549 | 24752266; 32645325 |
| BLM | Induced response | ND | HEK 293T, A549, Vero, MDCK | 26549460 |
| CTSC | Induced response | ND | Chicken (*Gallus spp*.) trachea/lung isolates | 19494054 |
| CD44 | Signature | Upregulation | PM | 21589892; 15972696 |
| CD86 | Pro-viral inflammation | Upregulation | Lung cancer; lung tissue | 19004938; 25144228 |
| CDK1^$^ | Upregulation, pro-viral | ND | PM | 27486199 |
| CNTRL | ND | Protein interaction | SARS-CoV-2 transcriptomics/ proteomics | 32353859 |
| CENPE^$^ | Differential expression | Altered expression | Hepatoma; A549, MDCK | 15858003; 32267861 |
| DYNLL1 | Viral mRNA interaction | Downregulation | Primary HBE; GISAID database | 32511382; https://doi.org/10.1101/597617 |
| EHMT2^$^ | Downregulation | ND | Embryonic kidney 293T; AHABE | 31034753 |
| **FGFR2** | Induced activation | ND | Alveolar epithelial | 31860803 |
| GAST | ND | Upregulation | Human fetal and pediatric stomach tissue | https://doi.org/10.1101/2020.06.24.167049 |
| GBA | Anti-viral regulation | ND | Embryonic & hamster (*mesocricetus spp.)* kidney, lung cancer | 30918081 |
| HDAC1^$^ | Anti-viral response | Pro-viral response | MDCK, AHABE various | 26912629; 32429325 |
| HIST1H1B^$^ | Increased protein abundance | ND | Mast | 31271652 |
| HIST1H2BC^$^ | Downregulation | ND | A549 | 28273165 |
| HIST1H2BN | Downregulation | ND | A549, HEK 293T, HBE | 20081832 |
| HLA-DRA | ND | Allelic pro-viral susceptibility | T-cell epitope | 32106567 |
| HLA-DRB1 | Allelic pro-viral susceptibility | Allelic pro-viral susceptibility | Plasma | 29315655; 19445991 |
| HLA-DRB5 | Peptide interaction | Candidate drug target | HLA-DR B cell lines, HTL, CTL, B-cell epitope | 8943576; 32376359 |
| HLA-DQB2 | Vaccine induced methylation | ND | PBMC | 27031986 |
| **IL2RA** | Altered expression | Upregulation | Bone marrow dendritic cells; Plasma | 21297642; 32470153 |
| IL7R^$^ | Inactivation | ND | Lung-resident memory T-cells | 28051085; 18097017 |
| IQGAP2 | Carbohydrate metabolism | ND | Airway epithelial cells | 22398282 |
| **KPNB1^$^** | Replication regulation | Downregulation | Epithelial; epithelial colorectal adenocarcinoma | 20027183; 21994648; 17596301 |
| MAPK10 | Upregulation | ND | PM | 27486199 |
| NTM | Downregulation | ND | Chicken (*Gallus spp*.) embryo fibroblasts | 22111699 |
| **NUP153^$^** | Degradation | ND | MDCK II; lung epithelial | 25810542 |
| NUP160 | Life cycle, transcription, replication; infection | ND | C127 | Corpus ID: 82630032; 2214032 |
| OSBPL10 | Viral signature | ND | PBMC | 21673802 |
| PPARG^$^ | Anti-viral | ND | HBEC; alveolar macrophages | 26248373; 30787149 |
| PIK3R2 | Pro-viral host interactions | ND |  | 20064372 |
| PPARGC1A | Upregulation | ND | HBE | 26437235 |
| PSMC3 | Viral protein | Upregulation | HBE | 28613140 |
| PSMD14 | Viral replication regulation | ND | ND | 20081832 |
| RARB | Downregulation | ND | A-MuLV-induced tumor; ascites | 25923039 |
| **STAT3** | Host cell resistance | Downregulation | MDKC, HEK 293T; Vero E6 kidney epithelial | 19327807; 15527783 |
| SLC15A1 | Variant interaction | ND | Pharyngeal and oropharyngeal swabs | 32015454 |
| SLC4A7^$^ | Downregulation | ND | Hippocampus | 29487124 |
| SPRED2 | Upregulation | ND | Lung tissue | 26757161 |
| ST6GALNAC3 | Variant interaction | ND | GIDEON database | 20174570 |
| TNS3 | Upregulation | ND | Postmortem mouse (*mus spp*.) lungs (Treg) | https://doi.org/10.1101/2020.06.05.135194 |
| TOLLIP | Pathogenicity regulation | ND | Lung adenocarcinoma cell line | 23300433 |
| TNRC6B | Upregulation | COVID-19 candidate drug target | miRDB, respiratory epithelial; GENEVESTIGATOR database | https://doi.org/10.21203/rs.3.rs-28866/v1; Genevisible** |
| UCHL5^$^ | Upregulation | ND | Postmortem mouse lungs | 23449804 |
| WDR48 | Viral signature | ND | Lung adenocarcinoma epithelial | 26738596; 31953534 |

**S2 Table.** **Human COVID-19 comorbidity-associated genes involved in influenza and/or SARS pathogenesis**

**^a^** COVID-19 comorbidities-associated genes identified by MAGMAv1.07b with direct involvement in influenza (*n* = 46) and/or SARS/COVID-19 (*n* = 17) are shown; **^b^** influenza involvement; **^c^** SARS involvement; **^d^** tissue or cell analyzed (all cell types are from human origin unless otherwise stated); **^e^** PubMed Identifier/reference; ^$^ genes in common with those found significant from a Gene Expression Omnibus (GEO) dataset for SARS using GEO2R (https://www.ncbi.nlm.nih.gov/geo/geo2r/?acc=GSE1739); * Influenza Research Database (IRD): comprehensive repository of publicly available influenza data (https://www.fludb.org/brc/hostFactorExperimentDetails.spg?method=SubmitForm&fromDetail=true&biosetIds=1057&expSeqId=149&resultMatrixUserDefId=ICL006_P_N_RM&decorator=influenza); ** Genevisible: most significant tissues, cell lines, diseases for gene of interest. (https://genevisible.com/perturbations/HS/Gene%20Symbol/TNRC6B).
