# Supplementary material for: Comorbidities and Susceptibility to COVID-19: A Generalized Gene Set Data Mining Approach
Source: J Clin Med. 2021 Apr 13;10(8):1666. doi: 10.3390/jcm10081666 (PMC8070572; doi:10.3390/jcm10081666)
Supplement: Supplementary file 1 [file jcm-10-01666-s001.zip › Revised Suppl. Files/Table S4 COVIDgenet Risk of Bias 04 08 21.docx]

**Table S3. Risk of bias assessment per PRISMA guideline**

|  | **Dorry et al, 2020** | **Konig et al, 2010** | **Mehle et al, 2010** | **Muhlbauer et al, 2015** | **Frieman et al, 2007** | **Mizutani et al, 2004** | **Jiang et al, 2019** | **Ando et al, 2011** | **Vasu et al, 2001** | **Le Sage et al, 2013** | **Tutuncuoglu et al, 2020** | **Shapira et al, 2009** | **König et al, 2010** | **Denisova et al, 2014** | **Bouhaddou et al, 2020** | **Domingues et al, 2015** | **Reemers et al, 2009** | **Lietzén et al, 2011** | **Chen et al, 2005** | **Yoshikawa et al, 2009** | **Moser et al, 2014** | **Söderholm et al, 2016** | **Gordon et al, 2020** | **Tang et al, 2005** | **Jiao et al, 2020** | **Ravindra et al, 2020** | **Ma et al, 2020** |
| --- | --- | --- | --- | --- | --- | --- | --- | --- | --- | --- | --- | --- | --- | --- | --- | --- | --- | --- | --- | --- | --- | --- | --- | --- | --- | --- | --- |
| **Random Sequence Generation** |  |  |  |  |  |  |  |  |  |  |  |  |  |  |  |  |  |  |  |  |  |  |  |  |  |  |  |
| **Allocation concealment** |  |  |  |  |  |  |  |  |  |  |  |  |  |  |  |  |  |  |  |  |  |  |  |  |  |  |  |
| **Blinding of participants and personnel** |  |  |  |  |  |  |  |  |  |  |  |  |  |  |  |  |  |  |  |  |  |  |  |  |  |  |  |
| **Blinding of outcome assessment** |  |  |  |  |  |  |  |  |  |  |  |  |  |  |  |  |  |  |  |  |  |  |  |  |  |  |  |
| **Incomplete outcome data** |  |  |  |  |  |  |  |  |  |  |  |  |  |  |  |  |  |  |  |  |  |  |  |  |  |  |  |
| **Selective reporting** |  |  |  |  |  |  |  |  |  |  |  |  |  |  |  |  |  |  |  |  |  |  |  |  |  |  |  |

**Table S4. Risk of bias assessment per PRISMA guideline (continued)**

|  | **Liu et al, 2019** | **Giobbe et al, 2020** | **Drews et al, 2019** | **Nagesh et al, 2016** | **Milazzo et al, 2020** | **Wu et al, 2019** | **Zhou et al, 2017** | **Karlas et al, 2010** | **Ahmed et al, 2020** | **Dutta et al, 2018** | **Keicho et al, 2009** | **Jurcevic et al, 1996** | **Kalita et al, 2020** | **Zimmermann et al, 2016** | **Sun et al, 2011** | **Ni et al, 2020** | **Zhou et al, 2017** | **Shen et al, 2008** | **Ioannidis et al, 2012** | **Kong et al, 2011** | **Huang et al, 1990** | **Lytkin et al, 2011** | **Traboulsi et al, 2015** | **Huang et al, 2019** | **Shapira et al, 2009** | **Kim et al, 2015** |
| --- | --- | --- | --- | --- | --- | --- | --- | --- | --- | --- | --- | --- | --- | --- | --- | --- | --- | --- | --- | --- | --- | --- | --- | --- | --- | --- |
| **Random Sequence Generation** |  |  |  |  |  |  |  |  |  |  |  |  |  |  |  |  |  |  |  |  |  |  |  |  |  |  |
| **Allocation concealment** |  |  |  |  |  |  |  |  |  |  |  |  |  |  |  |  |  |  |  |  |  |  |  |  |  |  |
| **Blinding of participants and personnel** |  |  |  |  |  |  |  |  |  |  |  |  |  |  |  |  |  |  |  |  |  |  |  |  |  |  |
| **Blinding of outcome assessment** |  |  |  |  |  |  |  |  |  |  |  |  |  |  |  |  |  |  |  |  |  |  |  |  |  |  |
| **Incomplete outcome data** |  |  |  |  |  |  |  |  |  |  |  |  |  |  |  |  |  |  |  |  |  |  |  |  |  |  |
| **Selective reporting** |  |  |  |  |  |  |  |  |  |  |  |  |  |  |  |  |  |  |  |  |  |  |  |  |  |  |

**Table S4. Risk of bias assessment per PRISMA guideline (continued)**

|  | **Leymarie et al, 2017** | **Wang et al, 2015** | **Sui et al, 2009** | **Bermúdez de León et al, 2020** | **Hosseini et al, 2018** | **Ito et al, 2016** | **Fumagalli et al, 2010** | **Morales-Nebreda et al, 2020** | **Schaefer et al, 2013** | **Haddad et al, 2020** | **Watanabe et al, 2013** | **Long et al, 2016** | **Hayward et al, 2020** |  |  |  |  |  |  |  |  |  |  |  |  |  |  |
| --- | --- | --- | --- | --- | --- | --- | --- | --- | --- | --- | --- | --- | --- | --- | --- | --- | --- | --- | --- | --- | --- | --- | --- | --- | --- | --- | --- |
| **Random Sequence Generation** |  |  |  |  |  |  |  |  |  |  |  |  |  |  |  |  |  |  |  |  |  |  |  |  |  |  |  |
| **Allocation concealment** |  |  |  |  |  |  |  |  |  |  |  |  |  |  |  |  |  |  |  |  |  |  |  |  |  |  |  |
| **Blinding of participants and personnel** |  |  |  |  |  |  |  |  |  |  |  |  |  |  |  |  |  |  |  |  |  |  |  |  |  |  |  |
| **Blinding of outcome assessment** |  |  |  |  |  |  |  |  |  |  |  |  |  |  |  |  |  |  |  |  |  |  |  |  |  |  |  |
| **Incomplete outcome data** |  |  |  |  |  |  |  |  |  |  |  |  |  |  |  |  |  |  |  |  |  |  |  |  |  |  |  |
| **Selective reporting** |  |  |  |  |  |  |  |  |  |  |  |  |  |  |  |  |  |  |  |  |  |  |  |  |  |  |  |

Risk of bias assessment via “Cochrane’s Handbook for Systematic Reviews of Interventions” [55] for 66 COVID manually selected articles confirming viral involvement with influenza/SARS from PubMed, DisGeNET, Influenza Research Database, and conventional Google searches where boxes in green depict low risk of bias, yellow depicts some risk of bias, and red depicts high risk of bias. Any text considered “abstract-only” or preprints were considered to have some concern of bias. One study was found to be at high risk of bias due to the full text being inaccessible, with the abstract only available to display results.
