# Supplementary figures and images for: Comorbidities and Susceptibility to COVID-19: A Generalized Gene Set Data Mining Approach
Source: J Clin Med. 2021 Apr 13;10(8):1666. doi: 10.3390/jcm10081666 (PMC8070572; doi:10.3390/jcm10081666)

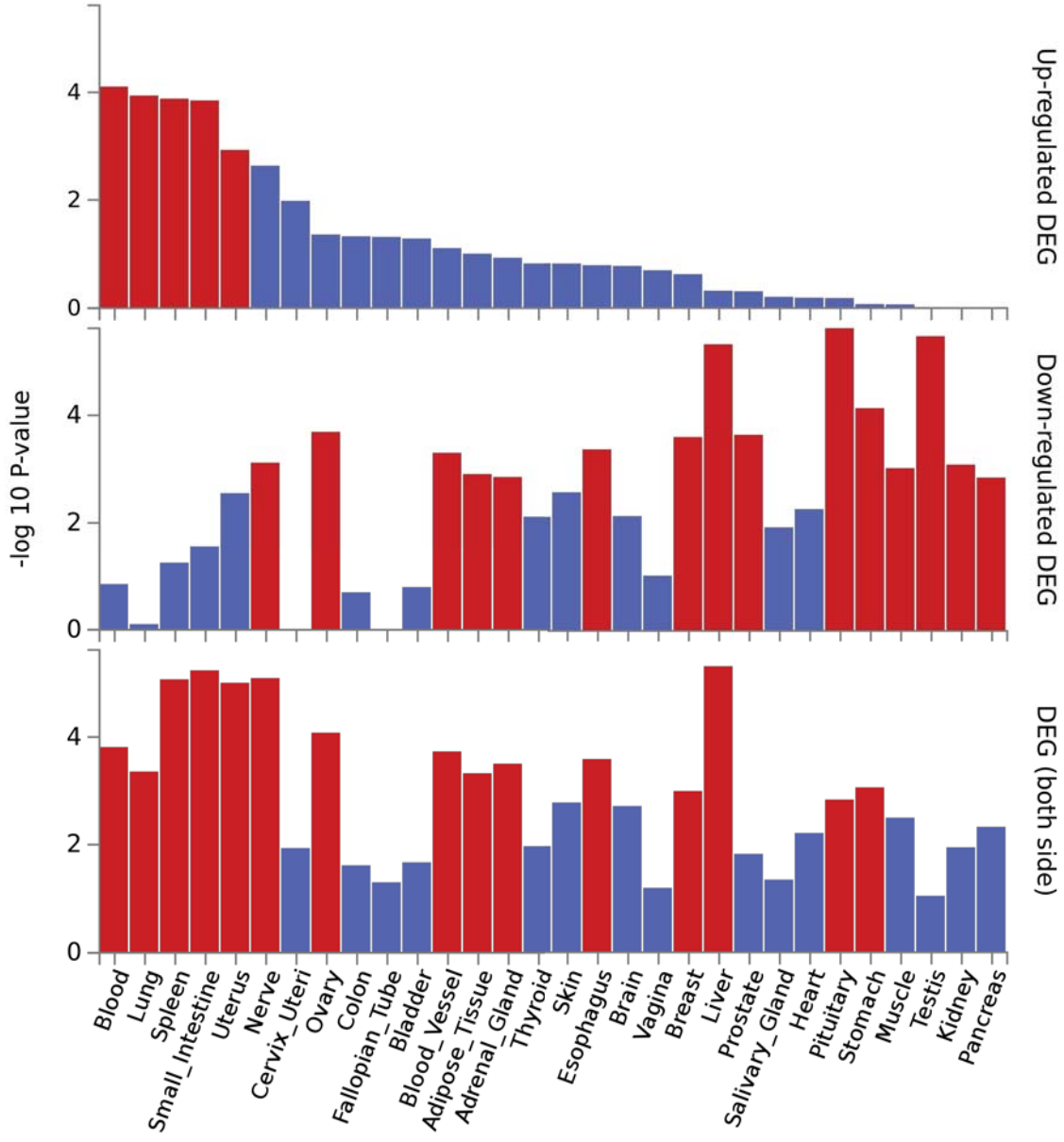

Supplement: Supplementary file 1 [file jcm-10-01666-s001.zip › Revised Suppl. Files/S2 Fig b COVIDgenet 54 tissues 02-05-2021.pdf]
